# Fisetin inhibits lipopolysaccharide-induced inflammatory response by activating β-catenin, leading to a decrease in endotoxic shock

**DOI:** 10.1038/s41598-021-87257-0

**Published:** 2021-04-16

**Authors:** Ilandarage Menu Neelaka Molagoda, Jayasingha Arachchige Chathuranga Chanaka Jayasingha, Yung Hyun Choi, Rajapaksha Gedara Prasad Tharanga Jayasooriya, Chang-Hee Kang, Gi-Young Kim

**Affiliations:** 1 Department of Marine Life Science, Jeju National University, Jeju, 63243 Republic of Korea; 2 Department of Biochemistry, College of Oriental Medicine, Dong-Eui University, Busan, 47227 Republic of Korea; 3 Department of Food Technology, Faculty of Technology, Rajarata University of Sri Lanka, Mihintale, 50300 Sri Lanka; 4 Bioresources Industrialization Support Department, Nakdonggang National Institute of Biological Resources, Sangju, 37242 Republic of Korea

**Keywords:** Biochemistry, Biophysics, Drug discovery, Immunology, Molecular medicine

## Abstract

Fisetin is a naturally occurring flavonoid that possesses several pharmacological benefits including anti-inflammatory activity. However, its precise anti-inflammatory mechanism is not clear. In the present study, we found that fisetin significantly inhibited the expression of proinflammatory mediators, such as nitric oxide (NO) and prostaglandin E_2_ (PGE_2_), and cytokines, such as interleukin-6 (IL-6) and tumor necrosis factor-alpha (TNF-α), in lipopolysaccharide (LPS)-stimulated RAW 264.7 macrophages. Additionally, fisetin attenuated LPS-induced mortality and abnormalities in zebrafish larvae and normalized the heart rate. Fisetin decreased the recruitment of macrophages and neutrophils to the LPS-microinjected inflammatory site in zebrafish larvae, concomitant with a significant downregulation of proinflammatory genes, such as *inducible NO synthase* (*iNOS*), *cyclooxygenase-2a* (*COX-2a*), *IL-6*, and *TNF-α*. Fisetin inhibited the nuclear localization of nuclear factor-kappa B (NF-κB), which reduced the expression of pro-inflammatory genes. Further, fisetin inactivated glycogen synthase kinase 3β (GSK-3β) via phosphorylation at Ser9, and inhibited the degradation of β-catenin, which consequently promoted the localization of β-catenin into the nucleus. The pharmacological inhibition of β-catenin with FH535 reversed the fisetin-induced anti-inflammatory activity and restored NF-κB activity, which indicated that fisetin-mediated activation of β-catenin results in the inhibition of LPS-induced NF-κB activity. In LPS-microinjected zebrafish larvae, FH535 promoted the migration of macrophages to the yolk sac and decreased resident neutrophil counts in the posterior blood island and induced high expression of *iNOS* and *COX-2a*, which was accompanied by the inhibition of fisetin-induced anti-inflammatory activity. Altogether, the current study confirmed that the dietary flavonoid, fisetin, inhibited LPS-induced inflammation and endotoxic shock through crosstalk between GSK-3β/β-catenin and the NF-κB signaling pathways.

## Introduction

Phagocytes, including macrophages and neutrophils, are the front-line immune defense cells that initiate inflammatory responses against invading pathogens^1^. However, bacterial endotoxins, including lipopolysaccharide (LPS) lead to dysregulated immune responses and promotion of tissue-damaging responses^1,2^. The chronic status of dysregulated immune responses is a significant cause of many inflammatory disorders, including cancer, cardiovascular diseases, and autoimmune diseases^2^.

LPS is an integral component of the outer membrane of gram-negative bacteria^3^. During infection, LPS is released, particularly, as a result of antibiotic treatment^4^. Previous studies have demonstrated that the toll-like receptor 4 (TLR4) signaling pathway is involved in LPS-induced inflammation^3,5^. In the intracellular domain of TLR4, myeloid differentiation primary response gene 88 (MyD88) activates the mitogen-activated protein kinase (MAPK) by recruiting interleukin-1 receptor-associated kinase 4 (IRAK-4) and tumor necrosis factor receptor-associated factor 6 (TRAF6), which subsequently activates the canonical inhibitor κB (IκB) kinase (IKK) to degrade IκBα. Ultimately, IκBα degradation promotes the nuclear translocation of free nuclear factor-kappa B (NF-κB) heterodimers, p50 and p65, thereby enhancing the transcriptional activities of NF-κB^6,7^. NF-κB is a well-characterized ubiquitous transcription factor that positively regulates the expression of inflammatory genes, such as *inducible nitric oxide synthase* (*iNOS*), *cyclooxygenase-2* (*COX-2*), *interleukin-6* (*IL-6*), and *tumor necrosis factor-α* (*TNF-α*)^8,9^. Therefore, targeting the NF-κB signaling pathway has been considered as a promising therapeutic strategy against LPS-induced inflammatory disorders^10^.

β-Catenin acts as a central mediator in intracellular signal transduction via the binding of extracellular Wnt to its receptors, such as frizzled (FZD) and low-density lipoprotein receptor- related protein (LRP)^11^. In the absence of Wnt, β-catenin is constitutively targeted for proteasomal degradation via the formation of a destructive complex with adenoma polyposis coli (APC), axin, glycogen synthase kinase-3β (GSK-3β), and casein kinase I^5,12^. In the presence of Wnt, GSK-3β is inactivated due to phosphorylation at Ser9, and thereby promoting β-catenin stabilization and nuclear translocation^13^. Although both the Wnt/β-catenin and NF-κB signaling pathways are conserved throughout mammalian development, the pathways independently regulate cell proliferation, cell survival, and cell differentiation. Nevertheless, a previous study on bacteria-colonized intestinal epithelial cells revealed that the overexpression of active β-catenin via GSK-3β inhibition reduced NF-κB activity and resulted in the downregulation of target inflammatory genes, thereby indicating that β-catenin downregulates the NF-κB-mediated inflammatory response^14^. Recently, Ma et al. confirmed that the crosstalk between Wnt/β-catenin and NF-κB positively or negatively modulates inflammation in a cell type- and/or gene-specific manner^15^, which suggests that NF-κB activation is noncanonically regulated through the Wnt/β-catenin signaling pathway. Agonists of the Wnt/β-catenin signaling pathway could thus serve as promising anti-inflammatory candidates in NF-κB-induced inflammatory disorders, such as septic shock.

Flavonoids exhibit many biological activities, including anti-inflammatory, anti-oxidant, anti-bacterial, and anti-allergic effects^16^. Among the bioactive flavonoids, fisetin (3,7,3′,4′-tetrahydroxyflavone) isolated from fruits and vegetables, including strawberry, apple, persimmon, grape, onion, and cucumber, possesses potent anti-oxidant and anti-cancer activities^17^. In addition, many previous studies have demonstrated that fisetin exerts its anti-inflammatory activity via inhibiting canonical NF-κB activation^18,19^. Recently, we found that fisetin directly binds to GSK-3β at non-ATP-binding sites, thereby promoting the nuclear localization of β-catenin via the inhibition of GSK-3β during melanogenesis^20^. Such findings indicated that fisetin could regulate noncanonical NF-κB activity via β-catenin activation. Nevertheless, the anti-inflammatory effect of fisetin against LPS-induced inflammation has not been studied. In the current study, we sought to determine whether fisetin inhibited LPS-induced inflammation in RAW 264.7 macrophages and endotoxic shock in zebrafish larvae via a crosstalk between GSK-3β/β-catenin and NF-κB pathways.

## Results

### High concentrations of fisetin decrease the viability of RAW 264.7 macrophages

**Figure 1:**
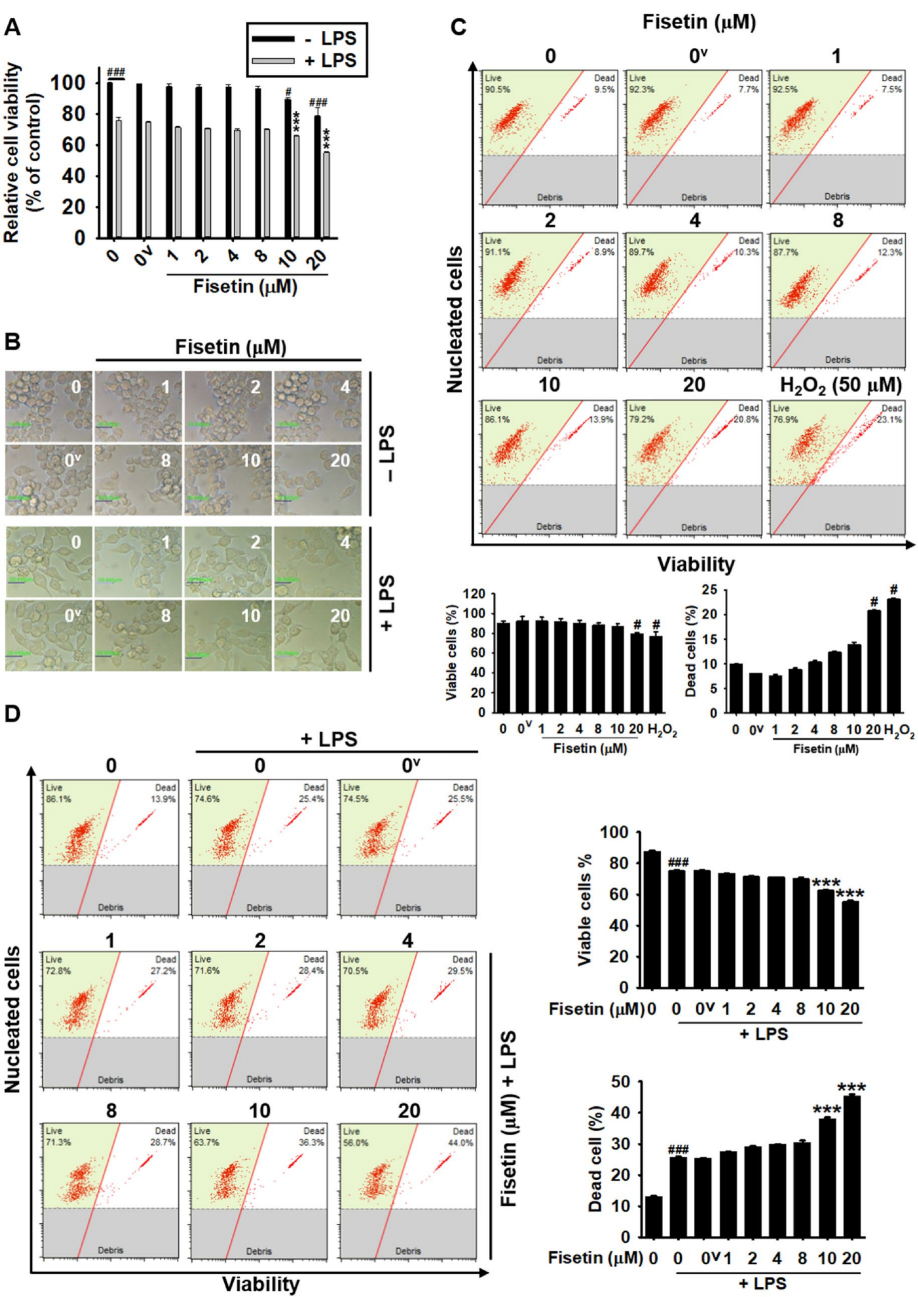
High concentrations of fisetin decreases the cell viability of RAW 264.7 macrophages. RAW 264.7 macrophages (1 × 10^5^ cells/mL) were treated with fisetin (0–20 µM) for 24 h in the presence or absence of 500 ng/mL LPS. (**A**) An MTT assay was performed. (**B**) Microscopic images was taken using phase contrast microcopy (× 10). Scale bares 40 μm. (**C**,**D**) Total viable cells and dead cells in the absence (**C**) and presence (**D**) of LPS were determined by flow cytometry. Each value indicates the mean ± SEM from three independent experiments. Significant differences among the groups were determined using an unpaired one-way ANOVA with Bonferroni correction. ^###^*p* < 0.001 and ^#^*p* < 0.05 vs. untreated cells; ***p < 0.001 vs. LPS-treated cells. 0^v^, vehicle control (0.1% DMSO).

To investigate the effect of fisetin on the viability of RAW 264.7 macrophages, the cells were treated with the indicated concentrations of fisetin for 24 h in the presence or absence of LPS. No significant change in cell viability was observed at concentrations of up to 8 µM fisetin compared to that of the untreated cells (97.8 ± 1.6%, 97.3 ± 1.7%, 97.5 ± 1.5%, 96.2 ± 1.9% at 1, 2, 4, and 8 µM fisetin); however, higher concentrations of fisetin significantly decreased the viability of RAW 264.7 macrophages (89.5 ± 1.3% and 78.8 ± 5.7% at 10 and 20 µM, respectively) (Fig. 1A). Additionally, LPS caused a decrease in cell viability (75.7 ± 2.4%) by potently inducing the differentiation of RAW 264.7 macrophages. Additionally, 10 µM and 20 µM fisetin further reduced cell viability in the presence of LPS (66.0 ± 0.4% and 55.2 ± 0.5%, respectively). However, cytotoxic hallmarks such as apoptotic bodies, floating cells, and cell debris were not visible at all concentrations of fisetin tested in this study under microscopy (Fig. 1B). To further confirm the fisetin-induced decrease in cell viability, the total percentages of viable cells and dead cells were measured using flow cytometry (Fig. 1C,D). These results aligned with those of the MTT assay, the percentage of total viable cells was 78.2 ± 1.5%, while that of dead cells was 21.4 ± 1.2%, at 20 µM fisetin (Fig. 1C), which are comparable to those of the H_2_O_2_-treated cells (positive control; H_2_O_2_ induces cell death). Furthermore, we found that LPS alone decreased the percentage of viable cell populations (75.0 ± 0.4%) and increased dead cell populations (25.0 ± 0.4%, Fig. 1D). Both 10 μM and 20 μM fisetin decreased viability to 62.28 ± 0.77% and 55.05 ± 0.98%, and increased dead cell populations up to 37.71 ± 0.77% and 44.95 ± 0.97%. Collectively, these results indicate that high concentrations of fisetin exhibit cytotoxicity in RAW 264.7 macrophages; however, cytotoxicity was not observed below a concentration of 8 µM.

### Fisetin inhibits LPS-induced proinflammatory mediators and cytokines in RAW 264.7 macrophages

**Figure 2:**
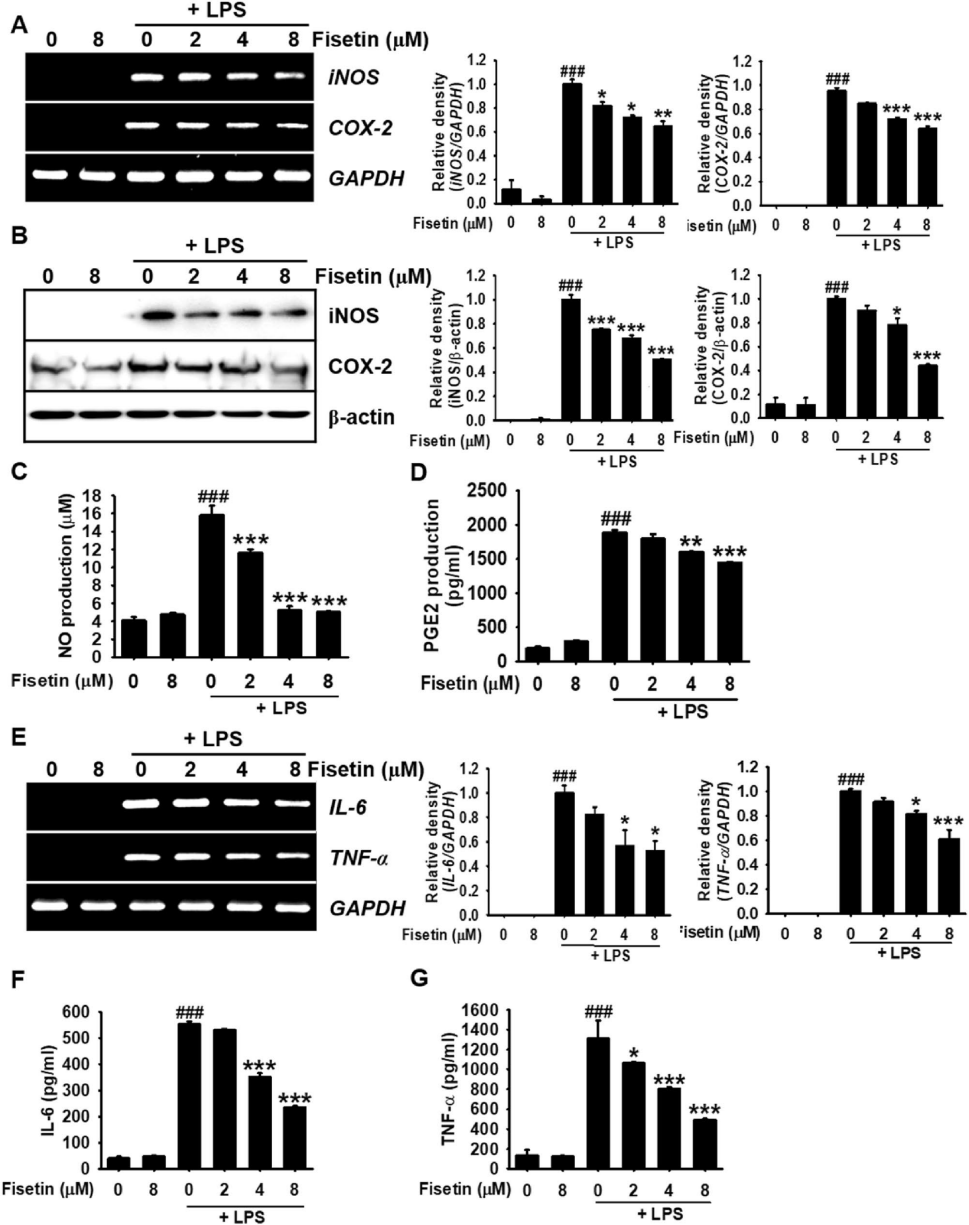
Fisetin decreases LPS-induced inflammatory mediator and cytokine levels in RAW 264.7 macrophages. RAW 264.7 macrophages (1 × 10^5^ cells/mL) were treated with fisetin (0–8 µM) 2 h before treatment with 500 ng/mL LPS. (**A**) Total mRNA was isolated at 6 h after 500 ng/mL LPS treatment, and RT-PCR was performed. *GAPDH* was used as an internal control. (**B**) Total proteins were isolated at 24 h and western blotting was performed. β-Actin was used as an internal control. (**C**) The amount of NO production in the culture medium was determined using the Griess Reagent Assay. (**D**) The amount of PGE_2_ was determined at 24 h using an ELISA according to the manufacturer’s instructions. (**E**) Total mRNA was isolated at 6 h and subjected to RT-PCR for *IL-6* and *TNF-α*. The amount of (**F**) IL-6 and (**G**) TNF-α was measured at 24 h by an ELISA. Each value indicates the mean ± SEM from three independent experiments. Significant differences among the groups were determined using the Student’s *t*-test (^###^*p* < 0.001 vs. untreated cells) and one-way ANOVA with Bonferroni correction (***p < 0.001, **p < 0.05, and *p < 0.01 vs. LPS-treated cells).

To confirm the anti-inflammatory effect of fisetin, RAW 264.7 macrophages were pretreated with fisetin for 2 h and stimulated with 500 ng/mL LPS for 6 h. RT-PCR revealed that LPS significantly increased *iNOS* and *COX-*2 expression; however, fisetin downregulated the LPS-induced expression of *iNOS* and *COX-*2 in a concentration-dependent manner (Fig. 2A). In agreement with this result, fisetin markedly downregulated LPS-induced iNOS and COX-2 protein expression by approximately twofold at a concentration of 8 µM (Fig. 2B). Additionally, NO production in LPS-stimulated macrophages was approximately fourfold (15.8 ± 0.4 µM) higher than that in the untreated cells (4.2 ± 0.4 µM). In contrast, pretreatment with fisetin reduced LPS-induced NO production in a concentration-dependent manner (11.7 ± 0.3 µM, 5.2 ± 0.5 µM, 5.1 ± 0.1 µM at 2, 4, and 8 µM fisetin, respectively) (Fig. 2C). Thereafter, fisetin was observed to decrease the release of PGE_2_ in the culture media in response to LPS treatment. ELISA revealed that LPS resulted in an approximately tenfold increase in PGE_2_ production (1888.7 ± 35.2 pg/mL) relative to that in the untreated cells (190. 7 ± 48.3 pg/mL) (Fig. 2D). Further, treatment with fisetin gradually weakened LPS-induced PGE_2_ production in a concentration-dependent manner (1796.1 ± 67.1 pg/mL, 1604.4 ± 6.5 pg/mL, 1445.8 ± 21.2 µM at 2, 4 and 8 µM fisetin, respectively) (Fig. 2D). In addition, fisetin was observed to drastically attenuate LPS-induced expression of *IL-6* and *TNF-α* at 6 h in a concentration-dependent manner (Fig. 2E). Accordingly, fisetin at a concentration of 8 µM significantly reduced LPS-induced IL-6 and TNF-α release up to 235.4 ± 4.9 pg/mL and 492.4 ± 11.7 pg/mL from 555.4 ± 7.4% pg/mL and 1310.7 ± 182.7% pg/mL, respectively (Fig. 2F,G). Furthermore, treatment effect of fisetin in LPS-induced inflammation was evaluated. We found that posttreatment with fisetin inhibited LPS-induced iNOS, COX-2, IL-6 and TNF-α in a concentration-dependent manner (Supplementary Fig. S1A) accompanied with the inhibitory levels of NO production (Supplementary Fig. S1B). Altogether, these results indicate that fisetin negatively modulates LPS-induced proinflammatory mediator and cytokine expression in RAW 264.7 macrophages.

### Fisetin attenuates mortality, abnormality, and lowered heart rate in LPS-microinjected zebrafish larvae

**Figure 3:**
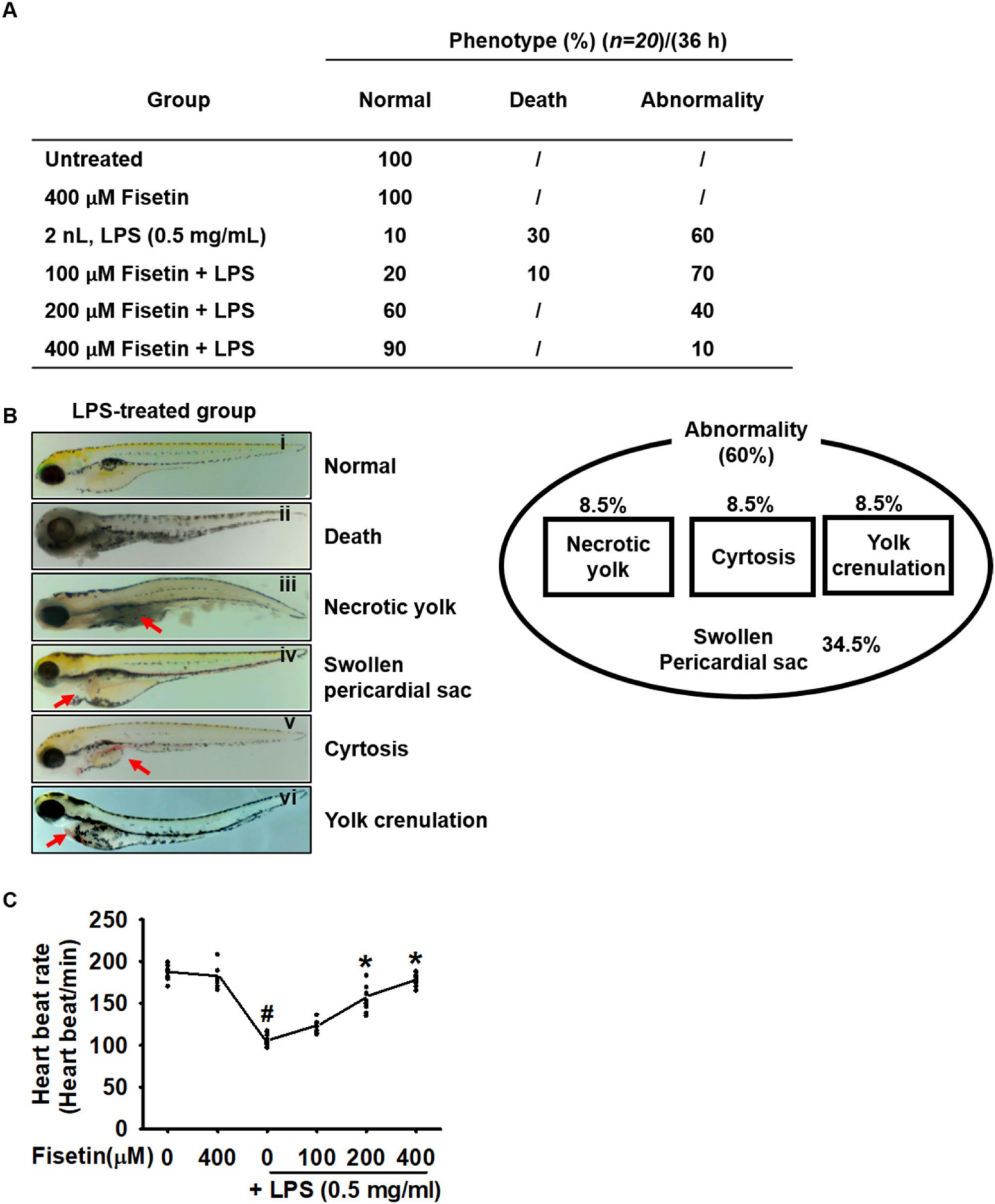
Fisetin attenuates LPS-induced mortality, abnormalities, and lowered heart rate in zebrafish larvae. Zebrafish larvae at 3 dpf (*n* = 20) were microinjected with 2 nL of 0.5 mg/mL LPS and immediately immersed in the indicated concentrations of fisetin. (**A**) Phenotype-based evaluations were performed at 36 h post injection (hpi). (**B**) LPS-microinjection increases abnormalities in zebrafish larvae at 36 hpi; this was observed using stereomicroscopy; (i) normal, (ii) death, (iii) necrotic yolk, (iv) swollen pericardial sac, (v) cyrtosis, and (vi) yolk crenulation. (**C**) LPS microinjection induces 60% abnormalities of zebrafish larvae and all larvae exhibits swollen pericardial sac (34.5%) either alone or together with necrotic yolk (8.5%), cyrtosis (8.5%), or yolk crenulation (8.5%). (**D**) Heart rates were measured to assess toxicity. Each value indicates the mean ± standard error median (SEM), and is representative of the results obtained from 20 fish for each group. Significant differences among the groups were determined using Student’s *t*-test (^#^*p* < 0.01 vs. untreated zebrafish larvae) and one-way ANOVA with Bonferroni correction (*p < 0.01 vs. LPS-treated zebrafish larvae).

To confirm that fisetin exhibits anti-inflammatory activity in an LPS-induced endotoxic shock model, 3 days post fertilized (dpf) zebrafish larvae were microinjected with LPS in the presence or absence of fisetin. Thereafter, mortality, abnormality (congenital malformations), and heart rate were measured. LPS-microinjection induced 30% death and 60% abnormality in zebrafish larvae at 36 dpf, and 10% normal survival (Fig. 3A). However, fisetin gradually decreased LPS microinjection-induced mortality to 10% at a concentration of 100 μM and caused complete blockage at concentrations ≥ 200 μM. Abnormality rates were 70%, 40%, and 10% at 100, 200, and 400 μM fisetin, respectively. In particular, fisetin-immersed larvae did not exhibit LPS-induced phenotypes, such as necrotic yolk, cyrtosis, and yolk crenulation—however, one (5%) did show a swollen pericardial sac—thereby indicating that fisetin attenuates LPS-induced severe endotoxic shock and phenotypes such as necrotic yolk. The morphological shapes of the LPS-microinjected larvae are shown in Fig. 3B: normal (1), death (2), necrotic yolk (3), swollen pericardial sac (4), cyrtosis (5), and yolk crenulation (6). We found that the swollen pericardial sac represents the dominant phenotype in all LPS-microinjected larvae. In brief, all LPS-microinjected abnormal larvae (60%) exhibited swollen pericardial sac, either alone (34.5%), or together with necrotic yolk (8.5%), cyrtosis (8.5%), and yolk crenulation (8.5%) (Fig. 3C). Additionally, we examined the heart rate of the larvae as an indicator of the toxicity evaluations. As shown in Fig. 3D, LPS microinjection resulted in a significant lowering of the heart rate (107 ± 6 heart beats/min) comparted to that in the PBS-microinjected larvae (185 ± 8 heart beats/min). Upon increasing the concentration of fisetin, the impaired heart rate gradually recovered almost up to the normal level at 400 µM (177.5 ± 6.9 heart beats/min). Altogether, these data indicate that fisetin attenuates LPS-induced septic shock in zebrafish larvae accompanied by decreased mortality, morphological abnormalities, and lowered heart rate.

### Fisetin inhibits LPS-induced proinflammatory gene expression and concomitantly decreases macrophage and neutrophil recruitment to the inflammatory sites in zebrafish larvae

**Figure 4:**
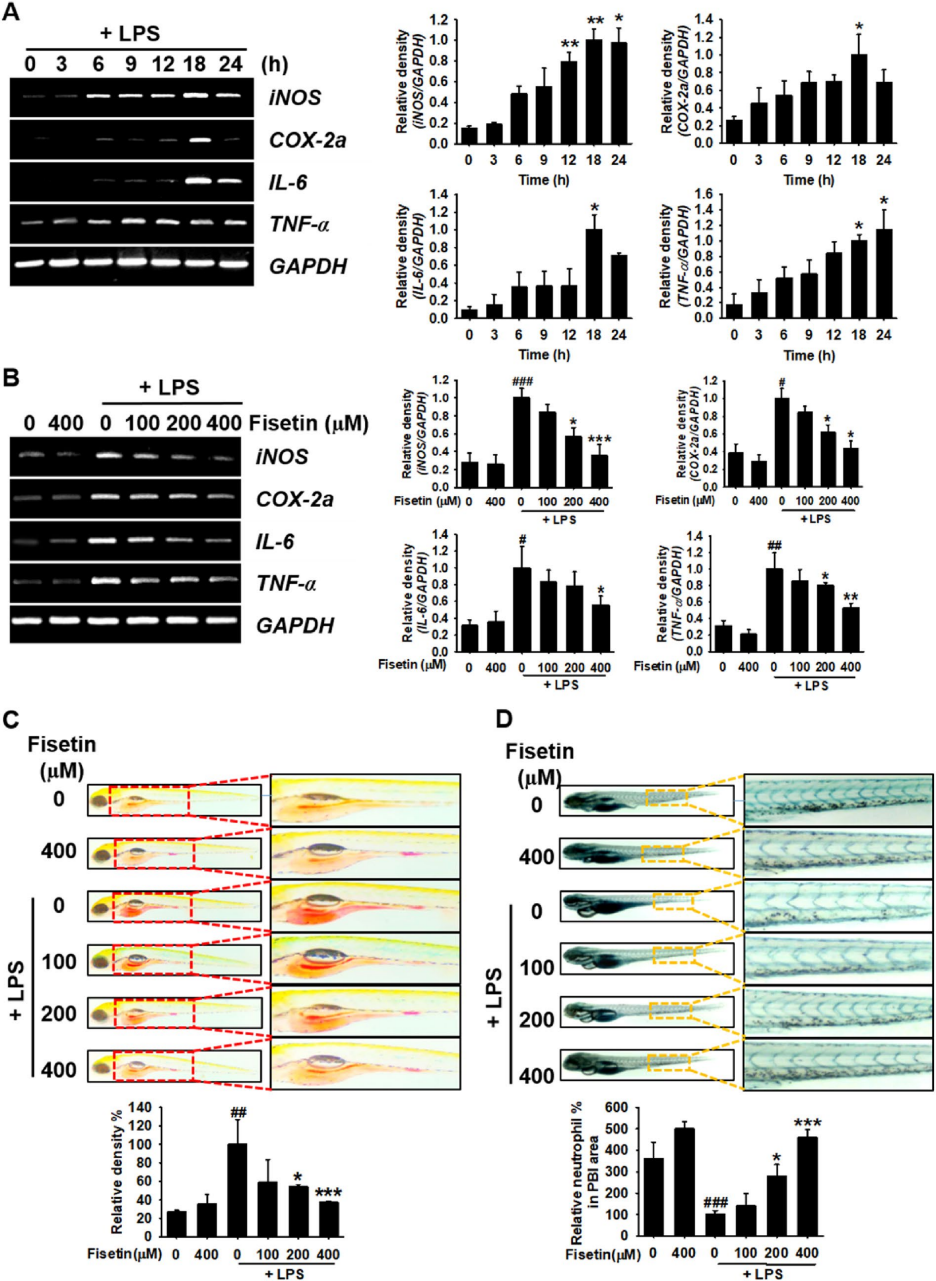
Fisetin inhibits LPS-induced inflammatory response in zebrafish larvae. Zebrafish larvae at 1 day post fertilization (dpf) were cultured in 0.003% PTU containing E3 embryo media. Briefly, 2 nL of 0.5 mg/mL LPS was microinjected into the yolk at 3 dpf. Zebrafish larvae were immediately immersed in E3 embryo media containing different concentrations of fisetin. (**A**) In LPS-microinjected conditions, 10 zebrafish were euthanized at the indicated time points and subjected to RT-PCR for evaluating the expression of *iNOS*, *COX-2a, IL-6*, and *TNF-α*. (**B**) At 18 h post injection (hpi), 20 zebrafish larvae from each treatment were euthanized and the expression of *iNOS*, *COX-2a, IL-6*, and *TNF-α* was measured by RT-PCR. (**C**) Neutral red staining of macrophages and (**D**) sudan black staining of the neutrophils were performed at 24 hpi. Each value indicates the mean ± standard error median (SEM) and is representative of the results obtained from three independent experiments. Significant differences among the groups were determined using the Student’s *t*-test (^###^*p* < 0.001, ^##^*p* < 0.01, and ^#^*p* < 0.05 vs. untreated zebrafish larvae) and one-way ANOVA with Bonferroni correction (***p < 0.001, **p < 0.01, and *p < 0.05 vs. LPS-treated zebrafish larvae).

To confirm whether fisetin inhibits the LPS-induced inflammatory response, 3 dpf zebrafish larvae were microinjected with LPS for 24 h. Thereafter, the time-dependent expression of proinflammatory mediators such as *iNOS* and *COX-2a* and cytokines such as *IL-6* and *TNF-α* was evaluated. In the LPS-microinjected condition, all genes tested in this study were expressed and reached maximal levels at 18 h, with a slightly different expression patterns (Fig. 4A). *iNOS* and *TNF-α* were significantly expressed at 6 h and their expression lasted for 24 h, whereas *COX-2a* and *IL-6* were highly expressed at 18 h. To evaluate the anti-inflammatory effect of fisetin in vivo, LPS-microinjected zebrafish larvae were grown in the presence of the indicated concentrations of fisetin for 18 h, and the expression level of the proinflammatory genes was detected. RT-PCR showed that fisetin concentration-dependently decreased the expression of *iNOS*, *COX-2a*, *IL-6*, and *TNF-α* in LPS-microinjected zebrafish larvae (Fig. 4B). In particular, a concentration of 400 µM fisetin was the potent at downregulating the expression of all proinflammatory genes tested in this study (i.e., the levels reached those in the untreated larvae). Furthermore, we sought to determine whether fisetin prevents the recruitment of macrophages and neutrophils to the inflammatory site in LPS-microinjected zebrafish larvae. Neutral red staining revealed that LPS injection significantly increased the macrophage counts at the site where LPS was injected (inflammatory site) in the yolk sac (red dot in the red box) at 24 h; however, immersion in fisetin resulted in a gradual decrease in the accumulation of macrophages in the yolk sac (Fig. 4C), indicating that fisetin inhibits the recruitment of macrophages from the circulating blood to the yolk sac, leading to the generation of anti-inflammatory responses. In alignment with the inhibition of macrophage recruitment, LPS-microinjection significantly decreased the large and clear cytolymph lipid droplets (accumulation of neutrophils, yellow dot box) in the posterior blood island (PBI) as neutrophils infiltrated the inflammatory site, i.e., the yolk sac (Fig. 4D). We also found that fisetin impaired the migration of neutrophils to the inflammatory site in a concentration-dependent manner, which indicates that fisetin attenuates the recruitment of neutrophils to the LPS-microinjected site. These results indicate that fisetin inhibits the LPS-induced inflammatory response by suppressing the expression of proinflammatory genes and reducing macrophage and neutrophil recruitment to the inflammatory sites.

### Fisetin inhibits LPS-induced NF-κB activity in RAW 264.7 macrophages

**Figure 5:**
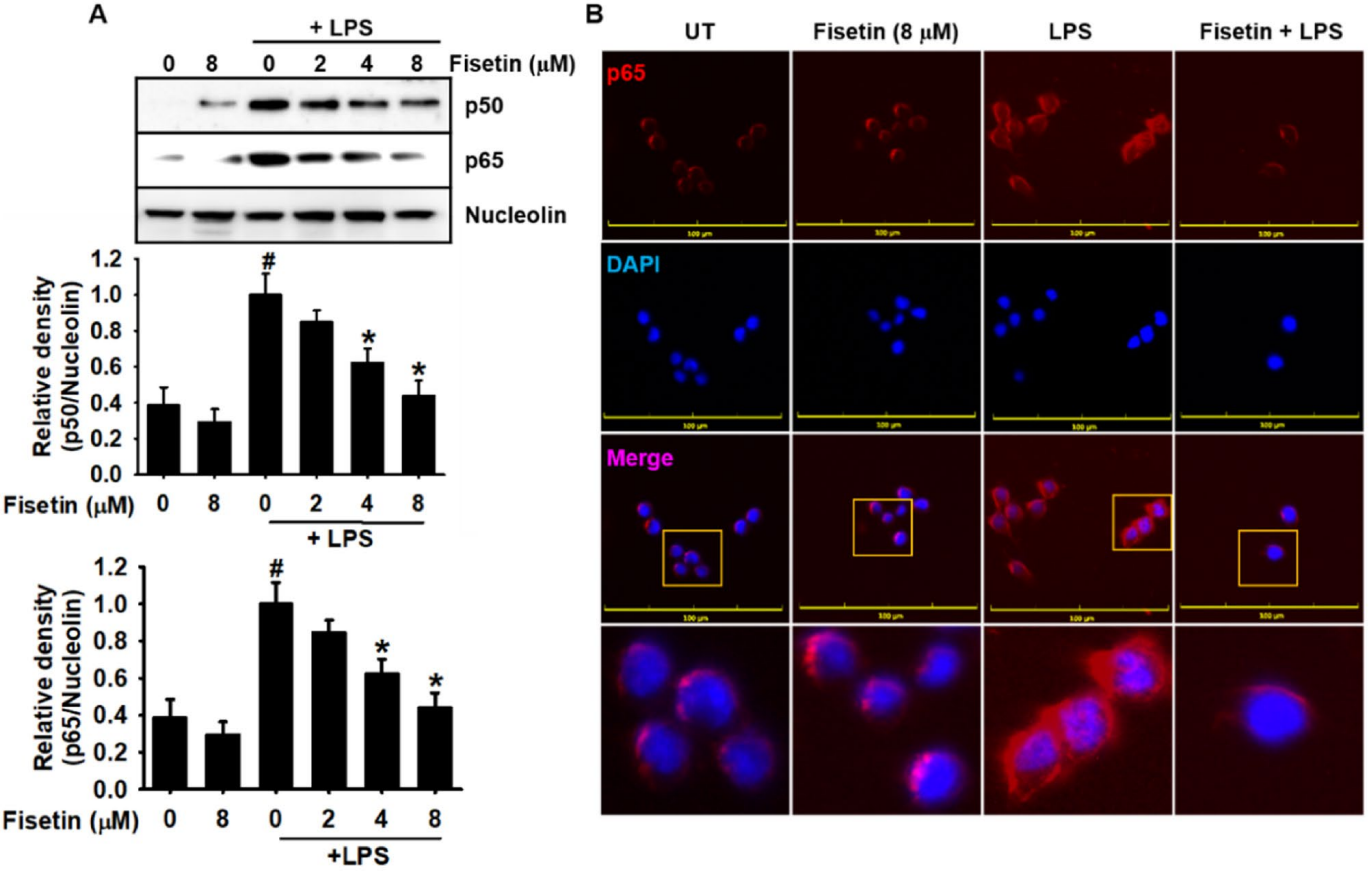
Fisetin inhibits LPS-induced nuclear translocation of NF-κB in RAW 264.7 macrophages. RAW 26.4.7 cells (1 × 10^5^ cells/mL) were pretreated with fisetin 2 h before LPS treatment (500 ng/mL; 0.5 h). (**A**) Nuclear proteins were isolated and equal amounts of proteins were resolved on SDS–polyacrylamide gels, transferred to PVDF membranes, and probed with antibodies against p50 and p65. Nucleolin was used as an internal control. (**B**) The nuclear translocation of p65 was detected by fluorescence microscopy. Each value indicates the mean ± standard error median (SEM), and is representative of the results obtained from three independent experiments. Significant differences among the groups were determined using Student’s *t*-test (^#^*p* < 0.05 vs. the untreated group) and one-way ANOVA with Bonferroni correction (*p < 0.05 vs. the LPS-treated cells). *UT* untreatment.

As NF-κB is considered as a major transcription factor in LPS-induced inflammatory response, we investigated whether fisetin negatively regulates the activation of the NF-κB pathway. Western blotting using nuclear extracts from RAW 264.7 macrophages confirmed that fisetin decreased LPS-induced nuclear localization of NF-κB p50 and p65 in a concentration-dependent manner (Fig. 5A). Additionally, immunohistochemistry confirmed that LPS rapidly stimulated the translocation of NF-κB p65 into the nucleus; however, fisetin inhibited the nuclear translocation of NF-κB p65 in the presence of LPS (Fig. 5B), indicating that fisetin inhibits LPS-induced NF-κB activation, resulting in the inhibition of inflammatory responses.

### Fisetin enhances phosphorylation of GSK-3β at Ser9 and subsequent activation of β-catenin in RAW 264.7 macrophages

**Figure 6:**
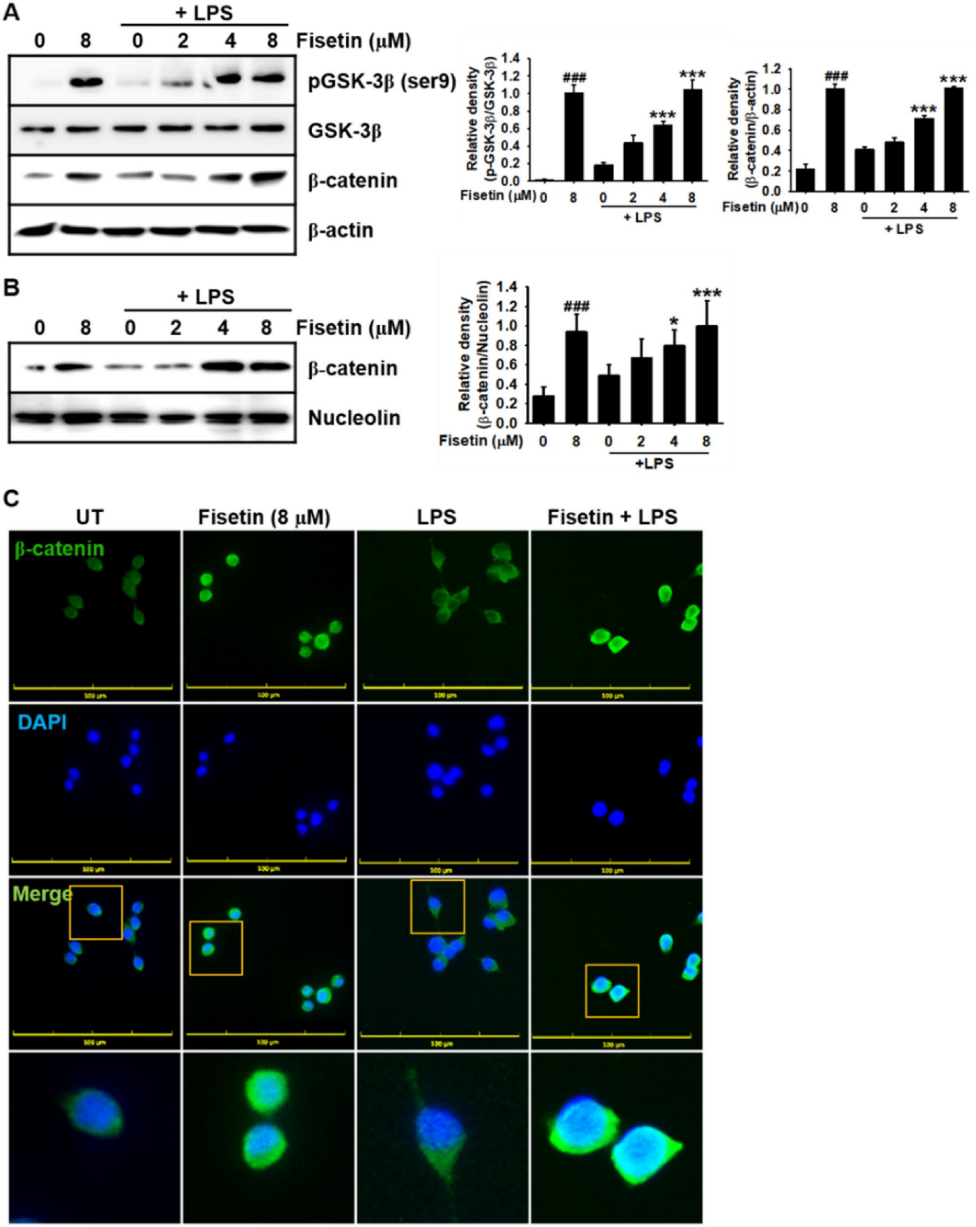
Fisetin promotes the expression of β-catenin and its nuclear translocation in LPS-stimulated RAW 264.7 macrophages. RAW 264.7 macrophages (1 × 10^5^ cells/mL) were pretreated with fisetin 2 h prior to LPS treatment (500 ng/mL; 0.5 h). (**A**) Total proteins were isolated and equal amounts of proteins were resolved on SDS–polyacrylamide gels, transferred to PVDF membranes, and probed with antibodies against total GSK-3β, phosphor-GSK-3β (Ser9), and β-catenin. β-Actin was used as an internal control. (**B**) In a parallel experiment, nuclear proteins were isolated and western blotting was performed to verify the nuclear translocation of β-catenin. Nucleolin was used as an internal control. (**C**) Nuclear translocation of β-catenin was detected by immunofluorescence. Each value indicates the mean ± standard error median (SEM), and is representative of the results obtained from three independent experiments. Significant differences among the groups were determined using Student’s *t*-test (^###^*p* < 0.001 vs. untreated cells) and one-way ANOVA with Bonferroni correction (***p < 0.001 and *p < 0.01 vs. LPS-treated cells). *UT* untreatment.

Recently, our research team revealed that fisetin binds to GSK-3β at non-ATP-binding site—through molecular docking prediction—and consequently activates β-catenin in B16F10 melanoma cells^20^. Deng et al. reported that β-catenin negatively regulates the inflammatory responses by inhibiting the expression of proinflammatory mediators and cytokines^14^. These data indicate that fisetin inhibits GSK-3β and subsequently stabilizes β-catenin, which attenuates LPS-induced inflammation. Therefore, we determined whether fisetin affects the expression of GSK-3β and β-catenin as well as the nuclear translocation of β-catenin. Fisetin concentration-dependently increased the phosphorylation of GSK-3β at Ser9—an inactive form—and enhanced the level of total β-catenin (Fig. 6A) and its nuclear translocation (Fig. 6B). Immunohistochemistry also revealed that fisetin enhanced the nuclear translocation of β-catenin regardless of the presence of LPS; however, an abundance of β-catenin was found in the cytosol after LPS treatment (Fig. 6C). Altogether, these results indicate that fisetin blocks the degradation of β-catenin by inhibiting GSK-3β, and consequently enhances the nuclear translocation of β-catenin.

### Fisetin inhibits β-catenin-mediated NF-κB activity, causing a significant decrease in LPS-induced IL-6 and TNF-α release

**Figure 7:**
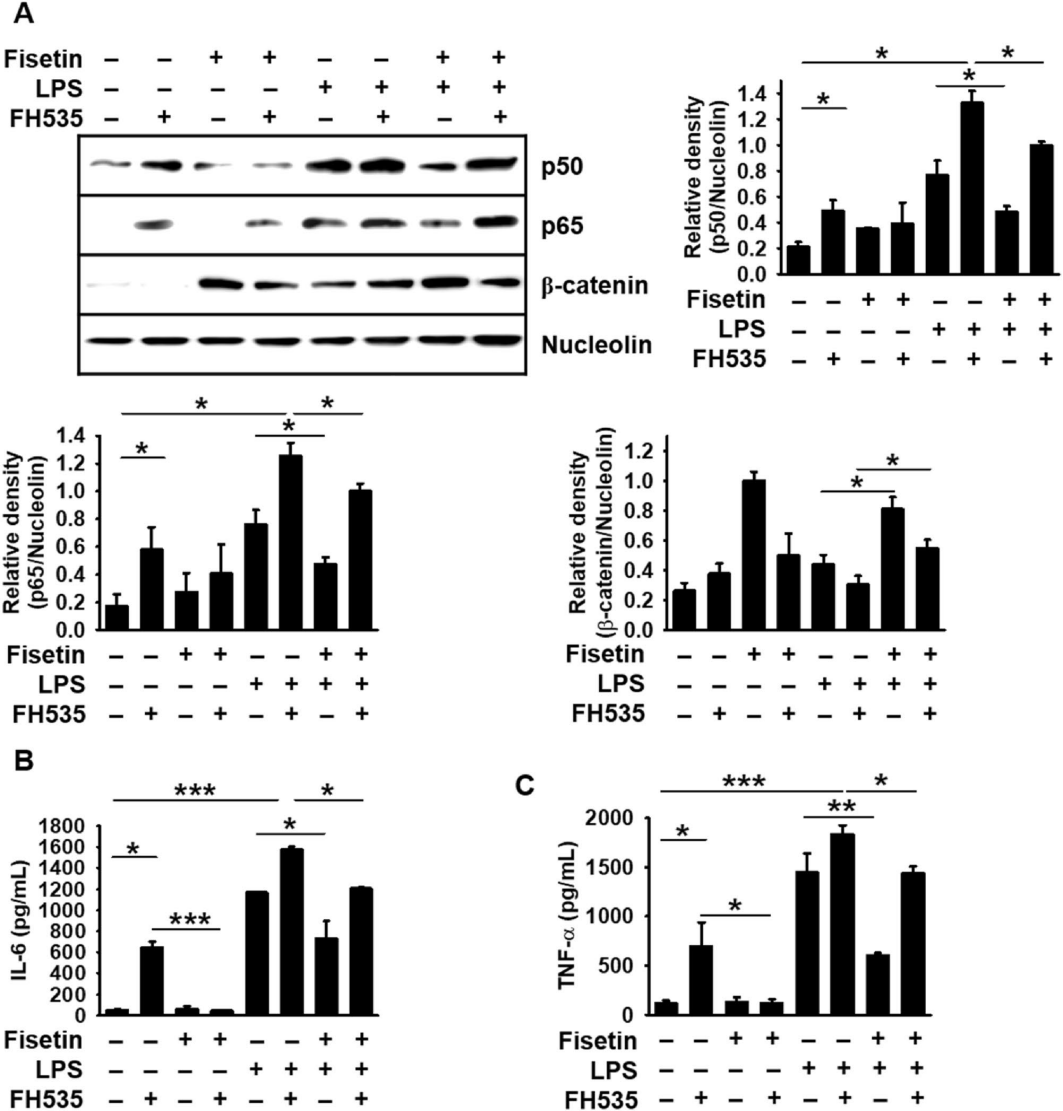
Fisetin inhibits LPS- or FH535-mediated IL-6 and TNF-α release by stimulating β-catenin-mediated NF-κB inactivation. RAW 264.7 macrophages (1 × 10^5^ cells/mL) were incubated with 10 µM FH535 for 2 h prior to treatment with 8 μM fisetin and 500 ng/mL LPS. (**A**) Nuclear proteins were extracted and western blotting was performed with specific antibodies against p65, p50, and β-catenin. Nucleolin was used as an internal control. (**B**,**C**) In a parallel experiment, the culture supernatants were collected and extracellular levels of (**B**) IL-6 and (**C**) TNF-α were measured by ELISA. Each value indicates the mean ± standard error median (SEM), and is representative of the results obtained from three independent experiments. Significant differences among the groups were determined using one-way ANOVA with Bonferroni correction (***p < 0.001, **p < 0.005, and *p < 0.01).

To confirm the crosstalk between β-catenin and NF-κB, RAW 264.7 macrophages were treated with FH535, an inhibitor of Wnt/β-catenin^21^, and LPS for 2 h prior to treatment with fisetin. FH535 itself considerably increased nuclear NF-κB p50 and p65 levels compared to those in the untreated cells, and moderately upregulated LPS-induced NF-κB p50 and p65 levels (Fig. 7A). In contrast, fisetin significantly inhibited the nuclear translocation of NF-κB p50 and p65 in the presence of FH535 or LPS, and combined treatment with FH535 and LPS-induced NF-κB p50 and p65 translocation to the nucleus was significantly inhibited in the presence of fisetin on (Fig. 7A). We also evaluated the effect of fisetin on LPS-induced IL-6 and TNF-α release in the presence of FH535. As shown in Fig. 7B,C, FH535 itself increased IL-6 (647.1 ± 53.6 pg/mL) and TNF-α (695.9 ± 243.3 pg/mL) release compared to those in the untreated cells (53.4 ± 10.4 pg/mL for IL-6 and 115.5 ± 29.6 pg/mL for TNF-α). Furthermore, simultaneous treatment with FH535 and LPS further elevated IL-6 (1577.9 ± 24.4 pg/mL) and TNF-α (1834.6 ± 91.8 pg/mL) levels compared to the LPS-treated condition (1170.1 ± 14.3 pg/mL and 1447.4 ± 36.8 pg/mL for IL-6 and TNF-α, respectively); however, fisetin significantly inhibited FH535-induced IL-6 (46.4 ± 2.5 pg/mL) and TNF-α (120.9 ± 36.8190 pg/mL) release. Additionally, fisetin significantly inhibited the IL-6 (1250.6 ± 11.8 pg/mL) and TNF-α (1437.7 ± 69.9 pg/mL) levels that were induced in response to combined treatment with LPS and FH535. Altogether, these results indicate that fisetin inhibits LPS-induced NF-κB activation in RAW 264.7 macrophages by activating β-catenin, which attenuates the expression of the proinflammatory cytokines, such as IL-6 and TNF-α.

### Fisetin-induced anti-inflammatory response is related to activation of β-catenin in an endotoxic shock model of zebrafish larvae

**Figure 8:**
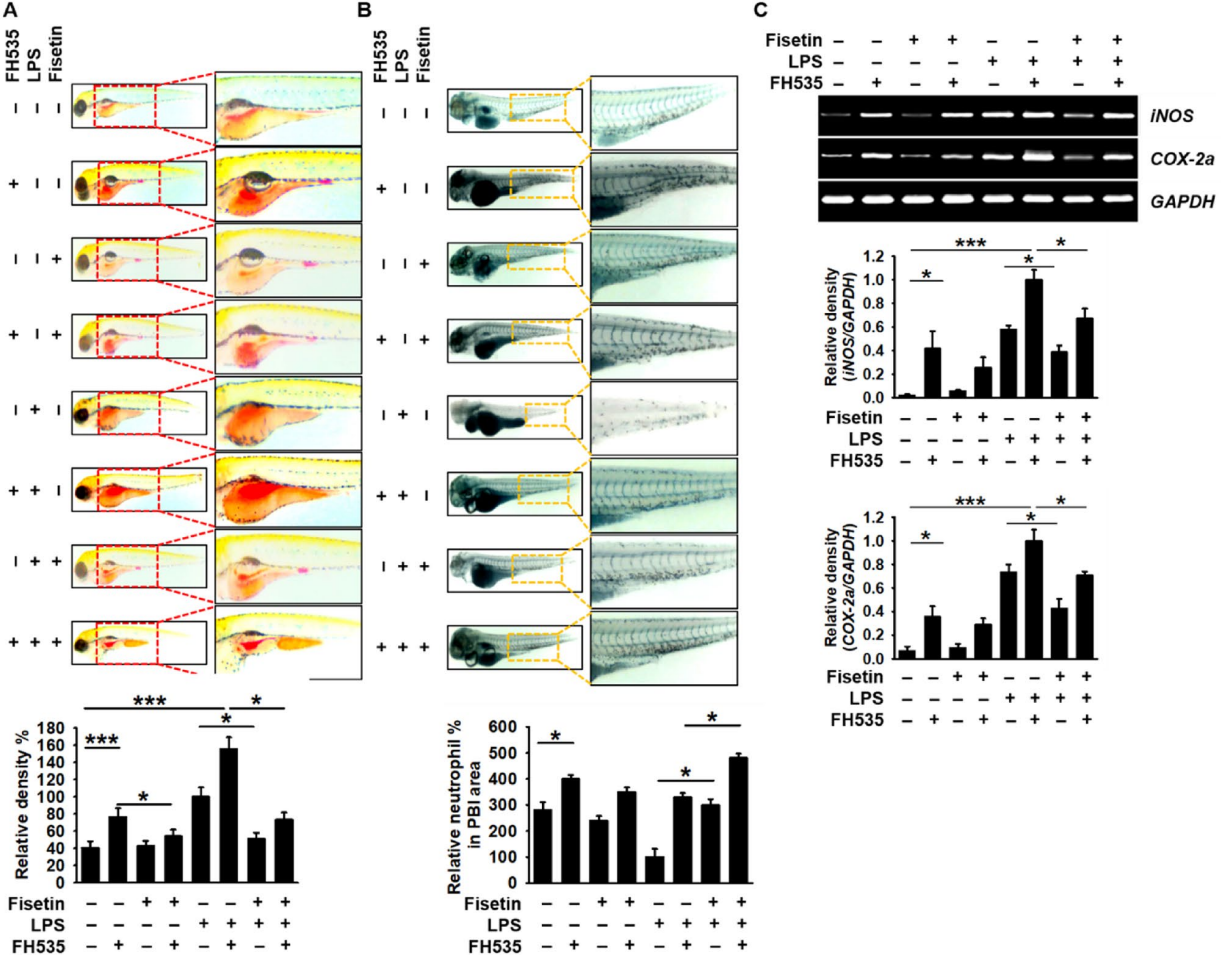
Fisetin inhibits LPS-induced inflammation and endotoxic shock by stimulating the β-catenin signaling pathway in zebrafish larvae. Zebrafish larvae at 3 dpf were pretreated with 10 µM FH535 for 2 h prior to LPS (2 nL of 0.5 mg/mL) microinjection. Then, the larvae were grown in E3 media containing 400 μM fisetin. (**A**) Neutral red-stained macrophages are shown at 18 hpi. (**B**) Sudan black-stained neutrophils were observed at 18 hpi. (**C**) At 18 hpi, 10 zebrafish from each condition were collected and the expression of *iNOS* and *COX-2a* was detected by RT-PCR. GAPDH was used as an internal control. Each value indicates the mean ± standard error median (SEM) and is representative of the results obtained from three independent experiments. Significant differences among the groups were determined using one-way ANOVA with Bonferroni correction (***p < 0.001, **p < 0.005, and *p < 0.01).

To confirm the significance of the β-catenin signaling pathway in fisetin-induced anti-inflammatory responses, we pharmacologically blocked the canonical β-catenin signaling pathway with FH535 in zebrafish larvae. We found that FH535 at 10 µM and 20 µM induced 20% and 100% mortality after 24-h of treatment. Further, 10 µM of FH535 increased the mortality up to 40% in LPS-microinjected larvae (data not shown). Interestingly, FH535-induced mortality was completely blocked in the presence of fisetin (data not shown), indicating that fisetin could reduce the mortality by activating the β-catenin signaling pathway. Thus, we investigated whether, in the presence of FH535, fisetin influences the recruitment of macrophages and neutrophils in LPS-microinjected zebrafish larvae. Interestingly, we found that FH535 by itself significantly increased the neutral red intensity (macrophages) in the yolk sac (Fig. 8A) and increased sudan black-stained spots (neutrophils) in the whole body, with neutrophils being retained at the PBI (Fig. 8B). As expected, in FH535-treated conditions, fisetin inhibited the recruitment of macrophages and neutrophils to the inflammatory site in LPS-microinjected zebrafish larvae, which indicated that fisetin-induced β-catenin activation hindered macrophage and neutrophil recruitment to the inflammatory sites. Subsequently, we investigated the expression of *iNOS* and *COX-2a* under the same experimental conditions. We found that both inflammatory genes were highly expressed in the presence of FH535 or LPS. Additionally, fisetin significantly inhibited FH535- or/and LPS-induced *iNOS* and *COX-2a* expression (Fig. 8C). These data imply that fisetin negatively regulates LPS-induced inflammation and endotoxic shock via activating the β-catenin signaling pathway.

## Discussion

**Figure 9:**
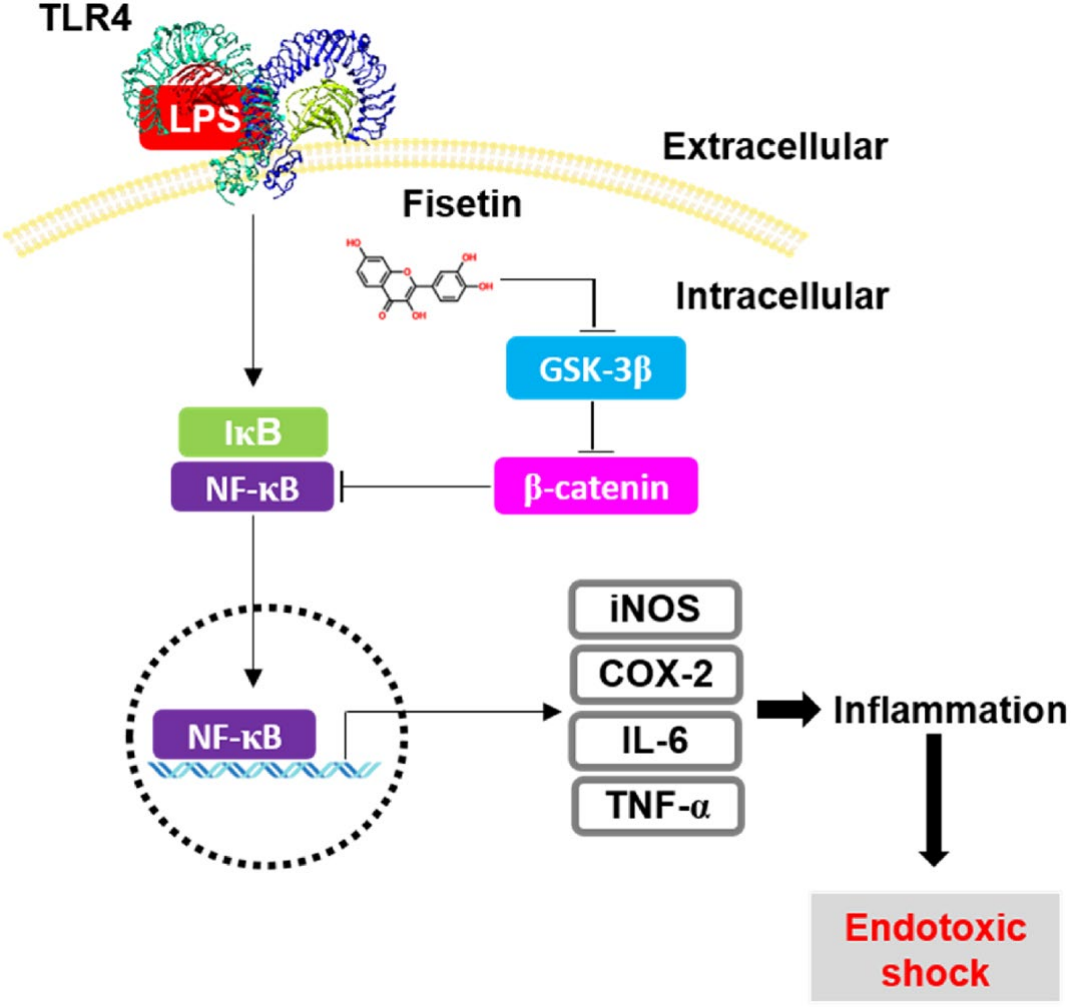
Fisetin inhibits GSK-3β-mediated NF-κB activation in the presence of β-catenin, leading to the inhibition of inflammation-induced septic shock. Once macrophages are exposed to high concentrations of the bacterial endotoxin, LPS, they initiate an inflammatory response and endotoxic shock by upregulating the expression of NF-κB-induced inflammatory genes, such as *iNOS, COX-2, IL-12, IL-6,* and *TNF-α*. Fisetin binds to the noncompetitive ATP-binding sites of GSK-3β and phosphorylates GSK-3β at Ser9, resulting in the inactivation GSK-3β and release of β-catenin from the destruction complex. The released β-catenin inhibits the transcriptional activity of NF-κB, thereby alleviating LPS-induced inflammation and endotoxic shock.

Endotoxic shock is a systemic inflammation accompanied by the excessive release of inflammatory mediators and cytokines, resulting in high cardiac output and mortality^22^. Non-steroidal anti-inflammatory drugs (NSAIDs), including aspirin, celecoxib, and diclofenac, are commonly used to combat systemic inflammation; however, they are associated with digestive problems such as upset stomach, heartburn, ulcers, and kidney injuries^23^. Therefore, small compounds, such as flavonoids, may provide a good alternative to prevent and reduce the risk of inflammation and endotoxic shock with relatively low side effects. Fisetin is a bioactive diphenyl propane flavone that is abundant in various plants. Its pharmacological properties including anti-cancer, antioxidant, and anti-inflammatory activity have been reported^16,18–20^. Nevertheless, the molecular mechanisms underlying the anti-inflammatory properties of fisetin are unclear. In the current study, we evaluated the anti-inflammatory effect of fisetin on LPS-induced inflammation and endotoxic shock in RAW 264.7 macrophages and zebrafish larvae. Our findings suggest that fisetin inhibits LPS-induced inflammation and endotoxic shock by suppressing β-catenin-mediated NF-κB activity, which subsequently attenuates the expression of proinflammatory mediators, such as NO and PGE_2_, and cytokines, such as IL-6 and TNF-α (Fig. 9).

Systemic inflammation is characterized by increase in proinflammatory cytokine levels, including IL-6, IL-12, and TNF-α, and other proinflammatory mediators, including NO and PGE_2_, via the activation of NF-κB^24–28^. Accumulating evidence on LPS-induced inflammatory disorders has revealed that LPS triggers the expression of inflammatory genes via the TLR4-dependent signaling pathway^3,5^. Binding of LPS to TLR4 leads to the phosphorylation of the IκB kinase (IKK) complex through the recruitment and activation of MyD88 and IRAK4, and in turn, phosphorylates IκB, which is degraded by the proteasome and allows the nuclear translocation of free NF-κB^6,7^. Therefore, targeting the NF-κB signaling pathway is thought to be a pivotal therapeutic strategy in the pathology of LPS-induced inflammatory disorders. Two research groups have previously reported that fisetin reduces ovalbumin-induced airway inflammation by inhibiting MyD88-mediated NF-κB activation^19,29^. In the present study, we reconfirmed that fisetin alleviated LPS-induced inflammatory mediator and cytokine levels in RAW 264.7 macrophages by inhibiting the NF-κB signaling pathway. Fisetin is also effective at protecting against metabolic dysfunction^30^, UVB^31^, cardiac ischemic injury^32^, and brain disorders^33^. Furthermore, fisetin inhibited LPS-induced macrophage activation and functional maturation of dendritic cells^34^ as well as LPS-induced acute lung injury^35^ by suppressing TLR4-mediated NF-κB signaling pathway. Nevertheless, whether the precious mechanism of fisetin-mediated endotoxic shock has not been elucidated. In the current study, using a zebrafish larva model, we proved that fisetin attenuated LPS-induced mortality and abnormality and caused a significant decrease in macrophage and neutrophil recruitment at the inflammatory site. Meanwhile, fisetin restored the heart rate up to the normal level along with the downregulation of proinflammatory genes during LPS stimulation, indicating that fisetin is a promising pharmacological candidate against LPS-induced endotoxic shock. Nevertheless, whether fisetin protects against endotoxic shock in preclinical studies is yet to be elucidated.

As the canonical Wnt/β-catenin signaling pathway plays a crucial role in the development of cancer through chronic inflammation, non-steroidal anti-inflammatory drugs, such as PPARγ agonists, might serve as potent therapeutic agents to treat cancers as they inhibit the Wnt/β-catenin pathway^36,37^. Alternatively, Swafford et al. reported that the deletion of Wnt receptors reduced histopathological severity and inflammation in the colon, along with high expression of anti-inflammatory cytokines such as IL-10, through the conditional activation of β-catenin^38^. Such findings suggest that the canonical Wnt/β-catenin pathway negatively regulates inflammation. Despite the dual regulation of Wnt/β-catenin in inflammation, in this study, fisetin increased total β-catenin and promoted its nuclear translocation in RAW 264.7 macrophages. Further, an increased expression of inactive GSK-3β (phosphorylated at Ser9) was observed. The inhibition of the Wnt/β-catenin signaling pathway by inhibitors of both Wnt/β-catenin and PPAR exacerbated the mortality and abnormality in zebrafish larvae. This was accompanied by a further accumulation of macrophages and neutrophils at the LPS-microinjected inflammatory site and a significant expression of proinflammatory genes. Interestingly, Deng et al. reported that β-catenin physically interacts with NF-κB to form a complex, and thereby reduces the DNA-binding ability of NF-κB^14^. Jun et al. also reported that gram-negative *Salmonella* infection constitutively activated β-catenin and thereby stabilized IκBα, which subsequently repressed the activity of NF-κB in HCT116 colon cancer cells^39^, indicating that the crosstalk between the Wnt/β-catenin and NF-κB pathways is linked to the regulation of inflammation. In agreement with the above studies, we found that fisetin-induced β-catenin attenuated LPS-induced inflammation and endotoxic shock by inhibiting NF-κB activation. Nevertheless, the discrepancy between the positive and negative regulatory role of the Wnt/β-catenin pathway in inflammatory disorders may be investigated in the context of NF-κB, even though NF-κB is a key activator of inflammation.

Our previous research predicted that fisetin directly binds to GSK-3β at the noncompetitive ATP-binding site and promotes β-catenin stabilization and nuclear translocation^20^. GSK-3β is a ubiquitous serine/threonine kinase involved in the molecular pathogenesis of severe disorders in humans, including inflammation, tumorigenesis, and neurological disorders^40^. In particular, Medunjanin et al. identified that GSK-3β directly phosphorylates NEMO, which is an essential activator of NF-κB and consequently activates NF-κB, indicating that GSK-3β stimulates a non-canonical NF-κB signaling pathway^41^. Ougolkov et al. also revealed that the inhibition or genetic depletion of GSK-3β inhibits NF-κB-induced gene transcription and subsequently leads to pancreatic cancer cell proliferation and survival by activating the NF-κB signaling pathway^42^. Based on our data, we deduced that the targeting of GSK-3β by fisetin inactivates the non-canonical NF-κB pathway and stabilizes β-catenin to inhibit LPS-induced inflammation and endotoxic shock. However, a recent study proposed the dual effect of GSK-3β on the anti-inflammatory and inflammatory response depending on the virulence factors, cell types, and physiological state of cells^43^. Therefore, the significance of the crosstalk between the GSK-3β/β-catenin and NF-κB signaling pathways needs to be persistently investigated in the pathogenesis of inflammatory disorders such as septic shock.

In conclusion, our findings suggest that fisetin attenuates LPS-induced inflammation and endotoxic shock by suppressing the β-catenin-mediated NF-κB signaling pathway. Fisetin can thus be considered as a potential anti-inflammatory drug for systemic inflammation.

## Materials and methods

### Reagents and antibodies

Fisetin, 3-(4,5-Dimethylthiazol-2-yl)-2,5-diphenyl-tetrazolium bromide (MTT), 1-phenyl-2-thiourea (PTU), and methylene blue were obtained from Sigma Chemical Co. (St. Louise, MO, USA). Mouse anti-human antibodies against iNOS (sc-7271), COX-2 (sc-19999), p50 (sc-8414), p65 (sc-8008), GSK-3β (sc-81462), phospho (p)-GSK-3β at Ser9 (sc-37800), β-catenin (sc-59737), β-actin (sc-69879), nucleolin (sc-13057), and peroxidase-labeled anti-mouse immunoglobulins were purchased from Santa Cruz Biotechnology (Santa Cruz, CA, USA). Peroxidase-labeled anti-rabbit antibody was purchased from Koma Biotechnology (Seoul, Republic of Korea). 2,5-Dichloro-N-(2-methyl-4-nitrophenyl) benzenesulfonamide (FH535) was obtained from Tocris (Bristol, UK). Dulbecco’s Modified Eagle Medium (DMEM), fetal bovine serum (FBS), antibiotic mixture, and trypsin-ethylenediaminetetraacetic acid (EDTA) solution were purchased from WELGENE (Gyeongsan, Gyeongsangbukdo, Republic of Korea). Alexa Fluor 488, Alexa Fluor 647, and goat anti-rabbit secondary antibody were purchased from Abcam (Cambridge, MA, UK). Dako faramount aqueous mounting solution was purchased from Dako (Carpinteria, CA, USA). All other chemicals were purchased from Sigma Chemical Co.

### Cell culture and viability^44,45^

RAW 264.7 macrophages were obtained from American Type Culture Collection (ATCC, Manassas, VA, USA) and cultured in DMEM supplemented with 5% FBS at 37℃ in 5% CO_2_. For the analysis of cell viability, the cells were seeded at a density 1 × 10^5^ cells/mL in 24 well plate and incubated with the indicated concentrations of fisetin. After 24-h incubation, MTT assay was performed.

### Flow cytometry analysis

RAW 264.7 macrophages were seeded at a density of 1 × 10^5^ cells/mL overnight and then treated with the indicated concentrations of fisetin for 24 h. After harvesting, the cells were washed with ice-cold phosphate-buffered saline (PBS) and incubated with Muse Cell Count and Viability Kit (Luminex Corp., Austin, TX, USA) for 10 min. Cell count and viability was measured by Muse Cell Cycler (Luminex Cop.).

### Isolation of total cellular RNA from RAW 264.7 macrophages and RT-PCR^44^

Total RNA was isolated from RAW 264.7 macrophages using easy-BLUE total RNA Extraction Kit (iNtRON Biotechnology, Seongnam, Gyeonggido, Republic of Korea) according to the manufacturer’s instruction. The RNA was reverse-transcribed by Moloney Murine Leukemia Virus (MMLV) Reverse Transcriptase Kit (Bioneer, Daejeon, Republic of Korea). In brief, synthetic cDNA was amplified using specific primers^44,46^.

### Western blot analysis^44^

Total cellular protein extracts were prepared by RIPA Lysis Buffer (iNtRON Biotechnology). The total protein lysates were centrifuged at 16,000*g* at 4 °C for 20 min. In a parallel experiment, cytoplasmic and nuclear proteins were prepared using NE-PER Nuclear and Cytosolic Extraction Reagents (Pierce, Rockford, IL, USA). Protein concentrations were measured by a Bio-Rad Protein Assay Kit (Bio-Rad, Hercules, CA, USA) and immediately used for western blotting.

### NO assay^44,47^

RAW 264.7 macrophage (1 × 10^5^ cells/mL) were seeded into 24-well plates and treated with the indicated concentrations of fisetin for 2 h prior to the stimulation with 500 ng/mL LPS for 24 h. Supernatants were collected and assayed for NO production by Griess reagent.

### Measurement of IL-6, TNF-α and PGE_2_

Enzyme linked immunosorbent assay (ELISA) kits were used to detect the expression levels of IL-6 (Thermo Fisher Scientific), TNF-α (BD Pharmingen, San Diego, CA, USA), and PGE_2_ (Cayman Chemicals, Ann Arbor, MI, USA) according to the manufacturer’s instructions. Briefly, RAW 264.7 macrophages (1 × 10^5^ cells/mL) were pretreated with the indicated concentrations of fisetin for 2 h prior to stimulation with 500 ng/mL LPS for 24 h. Supernatant was collected and used for each ELISA.

### Immunostaining^48^

RAW 264.7 macrophages (1 × 10^4^ cells/mL) were seeded on 3% gelatin-coated coverslips overnight and treated with the indicated concentrations of fisetin for 2 h prior to the exposure with LPS for 1 h. The cells were fixed with 4% paraformaldehyde (PFA) for 10 min at 37 °C, washed three times with ice-cold PBS, and permeabilized with 0.1% Triton X-100 for 10 min at room temperature, followed by washing with ice-cold PBS containing 0.1% tween 20 (PBST) for 5 min. The cells were blocked with 10% donkey serum and incubated with p65 and β-catenin antibody (1:100 in 10% donkey serum) overnight at 4 °C. After washing with ice-cold PBST, Alexa Fluor 488 and Alexa Fluor 647 secondary antibodies were added for p65 and β-catenin, respectively and incubated for 2 h at room temperature. For the counterstaining, the cells were incubated with DAPI (300 nM) for 10 min, washed three times with ice-cold PBST, and mounted with Dako Faramount Aqueous Mounting Media. Fluorescence images were captured by a CELENA S Digital Imaging System (Logos Biosystems, Anyang, Gyeonggido, Republic of Korea).

### Maintenance of zebrafish embryo and larvae

AB strain zebrafish were handled as previously described^49^. The zebrafish study was approved by Animal Care and Use Committee of Jeju National University (Jeju Special Self-governing Province, Republic of Korea; approval No.: 2020-0013). All methods were carried out in accordance with relevant guidelines and regulations. Additionally, all the methods were carried out in accordance with the ARRIVE guidelines^50^. Zebrafish were raised at 28.5℃ with a 14:10-h light:dark cycle in a water-recirculating tank system (pH 7.4 and 0.03% salinity). Fertilized embryos were collected after natural spawning and cultured at 28.5 °C in E3 embryo media containing 2 mg/L methylene blue. To inhibit melanin formation, 0.003% PTU was added to the egg water throughout the experimental period.

### LPS microinjection and cardiac toxicity evaluation

Three dpf zebrafish larvae were anesthetized using 0.04% tricaine and LPS (0.5 mg/mL, 2 nL in each larva) was microinjected into the yolk sac using Drummond NANOJECT III Injector (Drummond Scientific, Broomall, PA, USA). The negative control group was microinjected with PBS. After microinjection of LPS, the larvae were immediately placed in E3 media containing the indicated concentrations of fisetin. Dead larvae were removed within 0.5 hpi. Each group of larvae (*n* = 20) was cultured at 28.5 °C and observed for signs of phenotypic abnormality and mortality. The heart rate of the larvae was manually counted for one minute and used as an indicator for the cardiac toxicity evaluations. All mentioned parameters were observed using Olympus SZ2-ILST Stereomicroscopy (Tokyo, Japan).

### Neutral red staining^51^

Neutral red is a vital dye that accumulates in the lysosomes through endocytosis. As macrophage cells undergo efficient endocytosis, neutral red more robustly labels macrophages than any other cell types. Optimal staining of macrophages in live embryos was achieved by incubating embryos in 2.5 μg/mL neutral read solution containing 0.003% PTU at 28.5℃ in the dark for 6–8 h. After staining, macrophage migration was observed using Olympus SZ2-ILST Stereomicroscopy.

### Sudan black staining^51^

Sudan black is an azo stain that detects the presence of lipids with dark stains representing neutrophils. A stock solution of sudan black was prepared from sudan black powder (0.6 g) dissolved in pure ethanol (200 mL). A buffer solution was made from phenol (16 g) dissolved in pure ethanol (30 mL) plus Na_2_HPO_4_·12H_2_O (0.3 g) dissolved in distilled water (100 mL). A working staining solution was made by mixing stock solution (30 mL) with buffer (20 mL). Whole larvae were fixed with 4% methanol-free PFA in PBS for 2 h at room temperature and rinsed in PBS. The larvae were incubated in sudan black solution for 40 min, washed extensively in 70% ethanol, and then progressively rehydrated with PBS plus 0.1% Tween-20. The stained neutrophils were observed using Olympus SZ2-ILST stereomicroscopy.

### Isolation of total zebrafish mRNA and RT-PCR^52,53^

Total RNA was extracted from zebrafish larvae at 3-dpf which were injected with PBS or LPS (0.5 mg/mL) at the indicated time points (0–24 hpi). In a parallel experiment, LPS-microinjected zebrafish larvae were raised in the presence of fisetin and FH535 for 18 hpi. Total RNA was extract from the larvae using easy-BLUE Total RNA Extraction Kit (iNtRON Biotechnology). The RNA was reverse-transcribed by MMLV Reverse Transcriptase Kit (Bioneer) and synthetic cDNA was amplified using specific primers.

### Statistical analysis

RT-PCR and western blotting images were captured by ImageQuant LAS 500 (GE Healthcare Bio-Sciences AB, Uppsala, Sweden). All bands were shown a representative of three independent experiments and quantified by Image J Software (Wayne Rasband, National Institute of Health, Bethesda, MD, USA, www.imagej.net). The results shown in each of figure are a representative from three independent experiments. The data was fit with a modified three parameter exponential decay using SigmaPlot Version 12.0 (Systat Software, San Jose, CA, USA, www.systatsoftware.com). Significant differences between groups were determined using Student’s *t*-test or one-way ANOVA with Bonferroni correction. Values were presented as standard error of the mean (SEM). *** and ^###^*p* < 0.001, **p < 0.01, and *p < 0.05 were considered to indicate statistical significance.

## Supplementary Information

Supplementary Information
