# Supplementary material for: Fisetin inhibits lipopolysaccharide-induced inflammatory response by activating β-catenin, leading to a decrease in endotoxic shock
Source: Sci Rep. 2021 Apr 16;11:8377. doi: 10.1038/s41598-021-87257-0 (PMC8052411; doi:10.1038/s41598-021-87257-0)
Supplement: Supplementary file 1 — Supplementary Information [file 41598_2021_87257_MOESM1_ESM.docx]

**Fisetin inhibits lipopolysaccharide-induced inflammatory response by activating β-catenin, leading to a decrease in endotoxic shock**

**Ilandarage Menu Neelaka Molagoda^1^, Jayasingha Arachchige Chathuranga Chanaka Jayasingha^1^, Yung Hyun Choi^2^, Rajapaksha Gedara Prasad Tharanga Jayasooriya^3^,** **Chang-Hee Kang^4^**^✉^ **& Gi-Young Kim^1^**^✉^

^1^Department of Marine Life Science, Jeju National University, Jeju 63243, Republic of Korea

^2^Department of Biochemistry, College of Oriental Medicine, Dong-Eui University, Busan 47227, Republic of Korea

^3^Department of Food Technology, Faculty of Technology, Rajarata University of Sri Lanka, Mihintale 50300, Sri Lanka

^4^Bioresources Industrialization Support Department, Nakdonggang National Institute of Biological Resources, Sangju 37242, Republic of Korea

^✉^ e-mail: ckdgml3735@nnibr.re.kr (C.-H.K.) & immunkim@jejunu.ac.kr (G.-Y.K.)

**
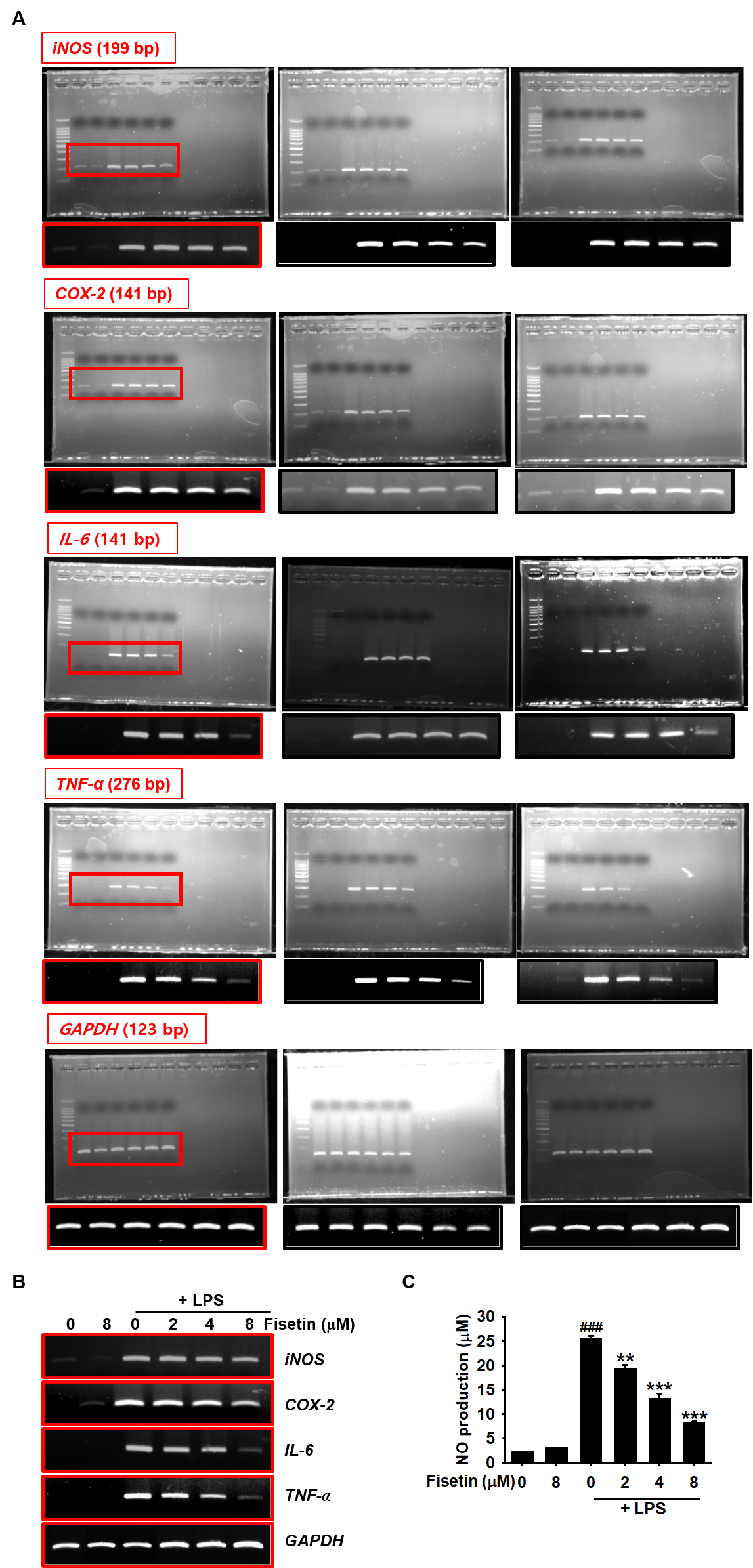
**

**Figure S1.** Fisetin decreases inflammatory responses in RAW 264.7 macrophages under LPS pretreated conditions. RAW 264.7 macrophages (1 × 10^5^ cells/mL) were pretreated with 500 ng/mL LPS 2 h before incubated with the indicated concentrations of fisetin (A) Total mRNA was isolated at 6 h after fisetin treatment, and RT-PCR was performed to analyze the expression of *iNOS, COX-2, IL-6 and TNF-α*. *GAPDH* was used as an internal control. (B) Reprehensive RT-PCR data were shown. (C)The amount of NO production in the culture medium was determined using the Griess assay.


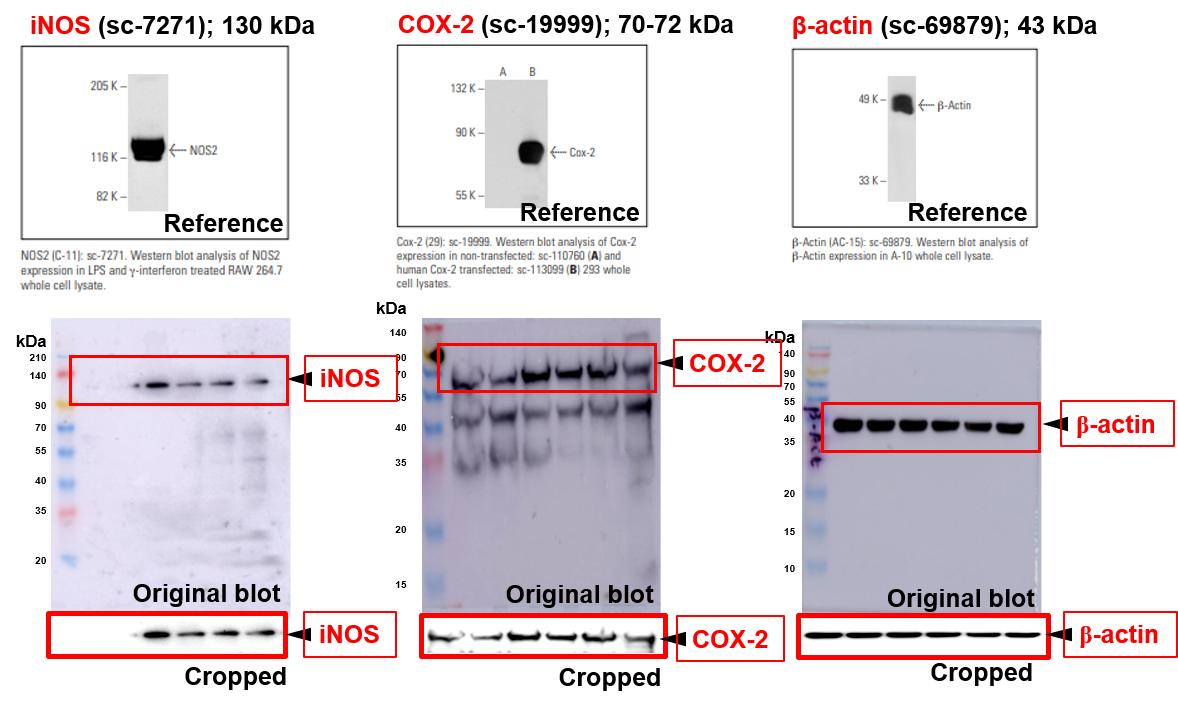


**Figure S2.** Uncropped images of Figure 2B.


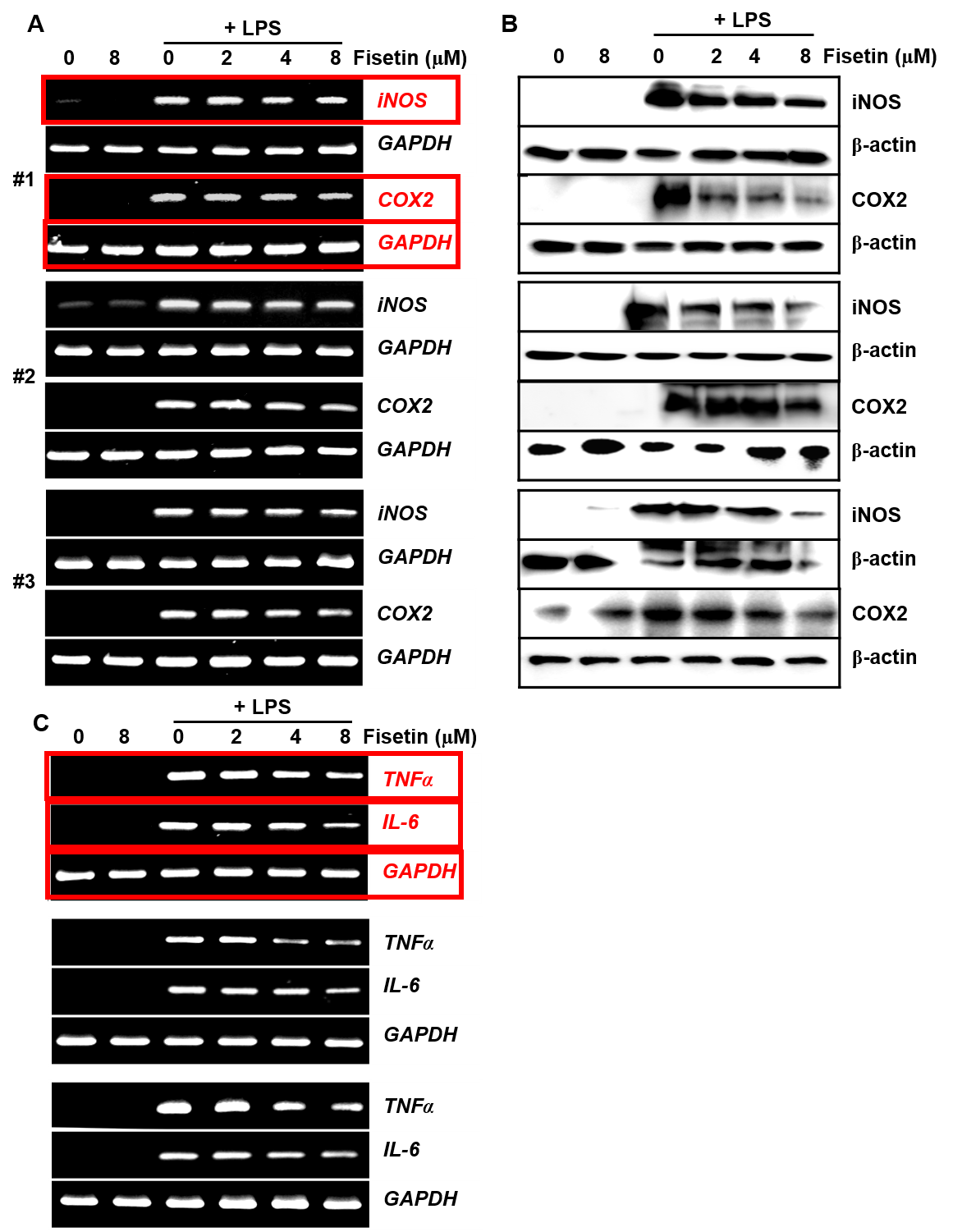


**Figure S3.** (A) Triplicated images of Figure 2A. (B) Triplicated images of Figure 2B. (C) Triplicated images of Figure 2E.


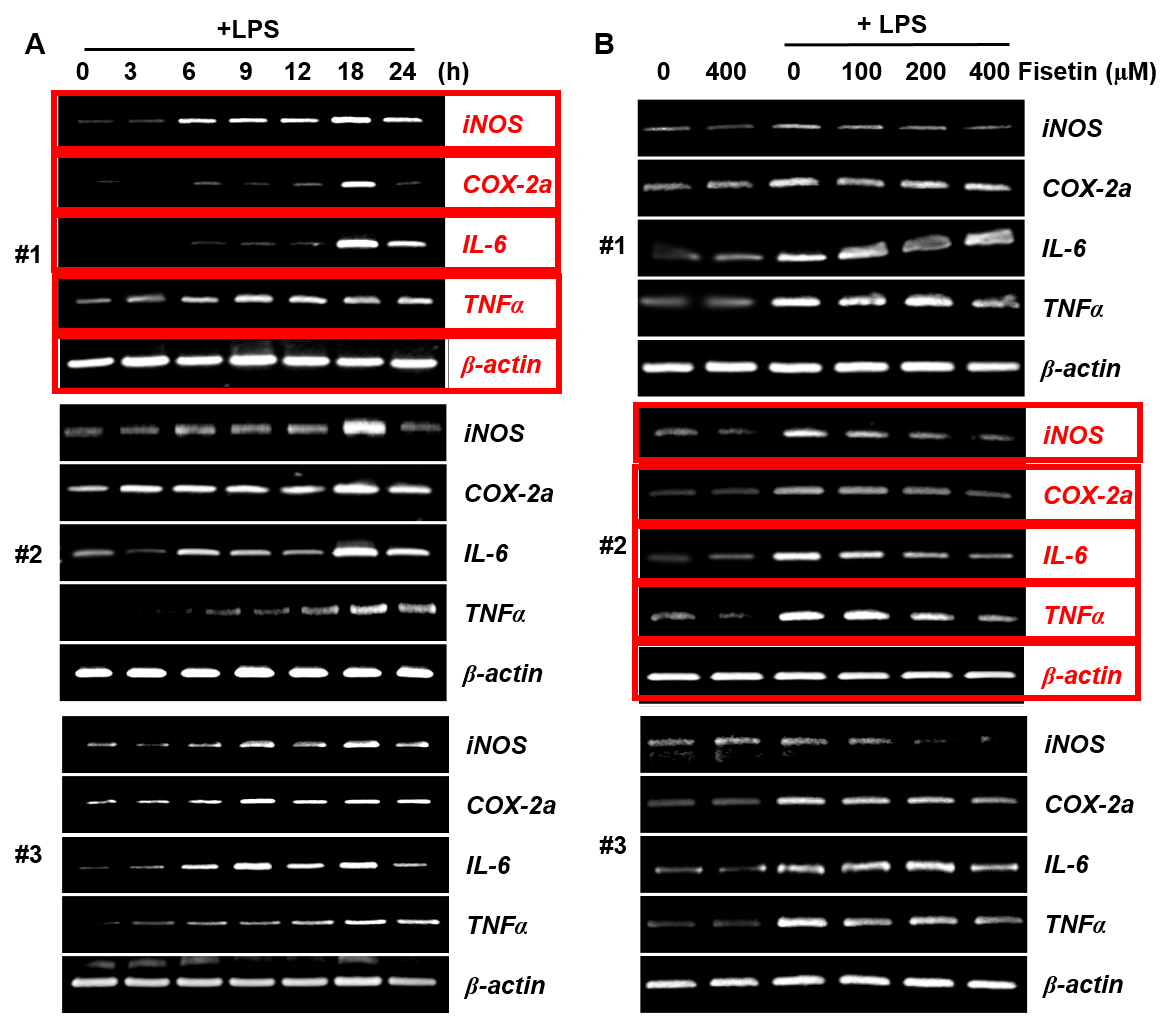


**Figure S4.** Cropped and triplicated images of Figure 4A and Figure 4B.


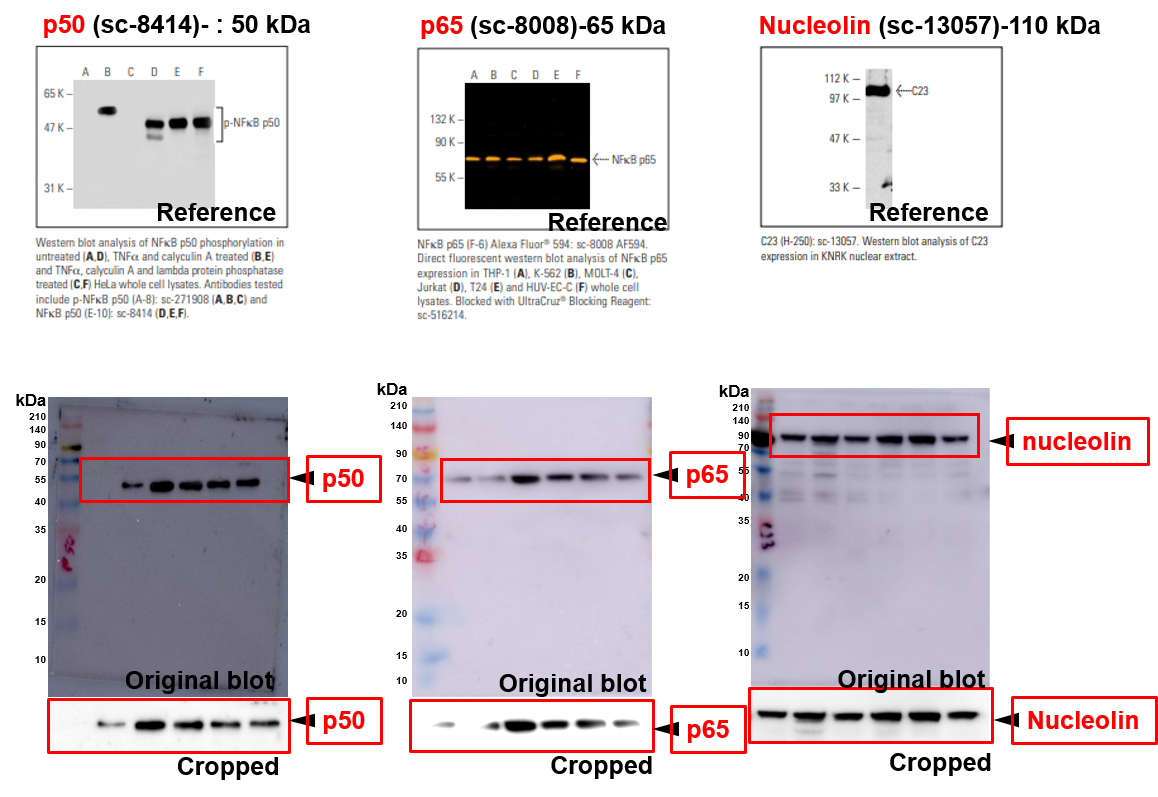


**Figure S5.** Uncropped images of Figure 5A.


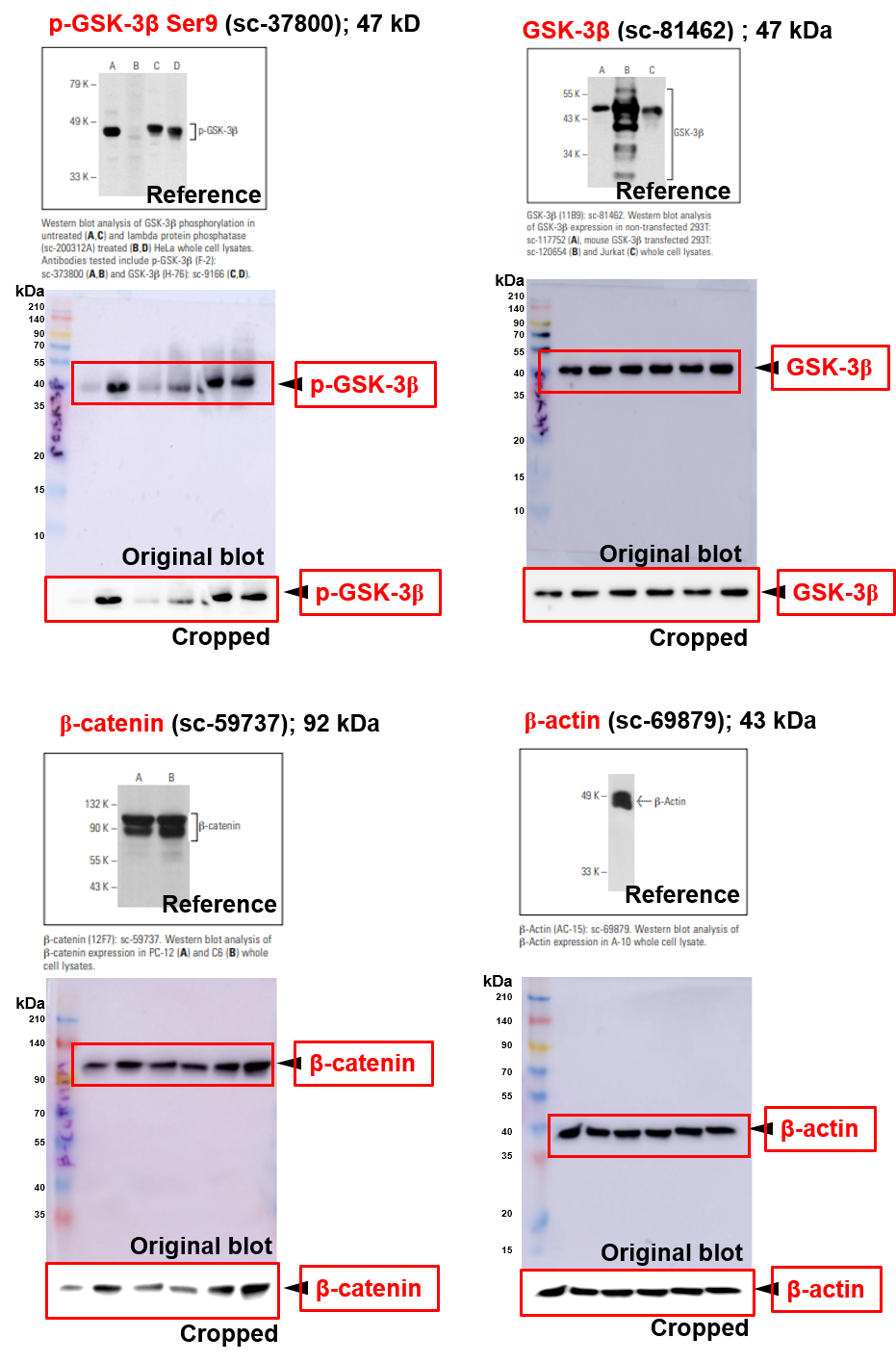


**Figure S6.** Uncropped images of Figure 6A.


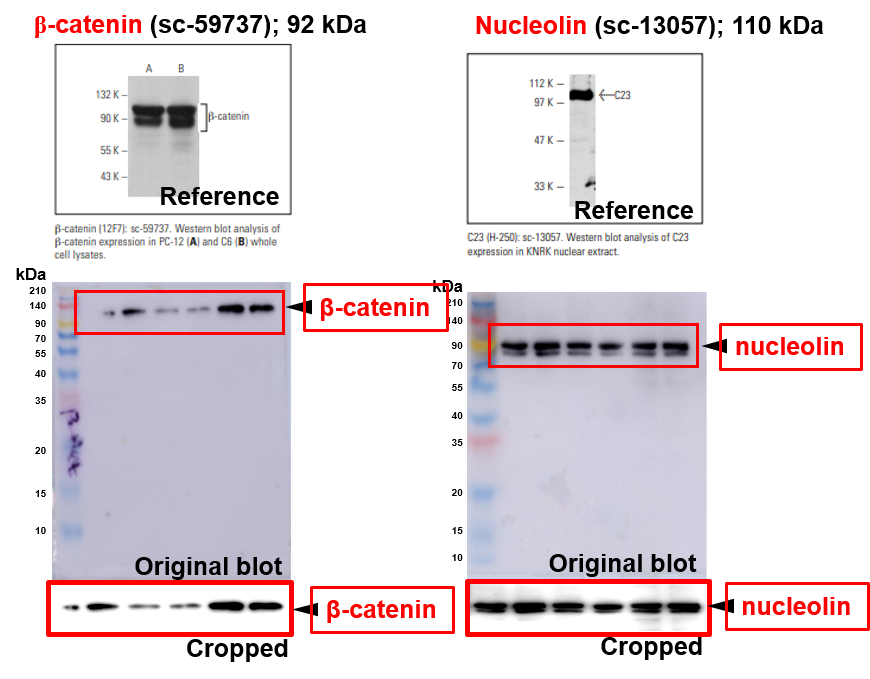


**Figure S7.** Uncropped images of Figure 6B.


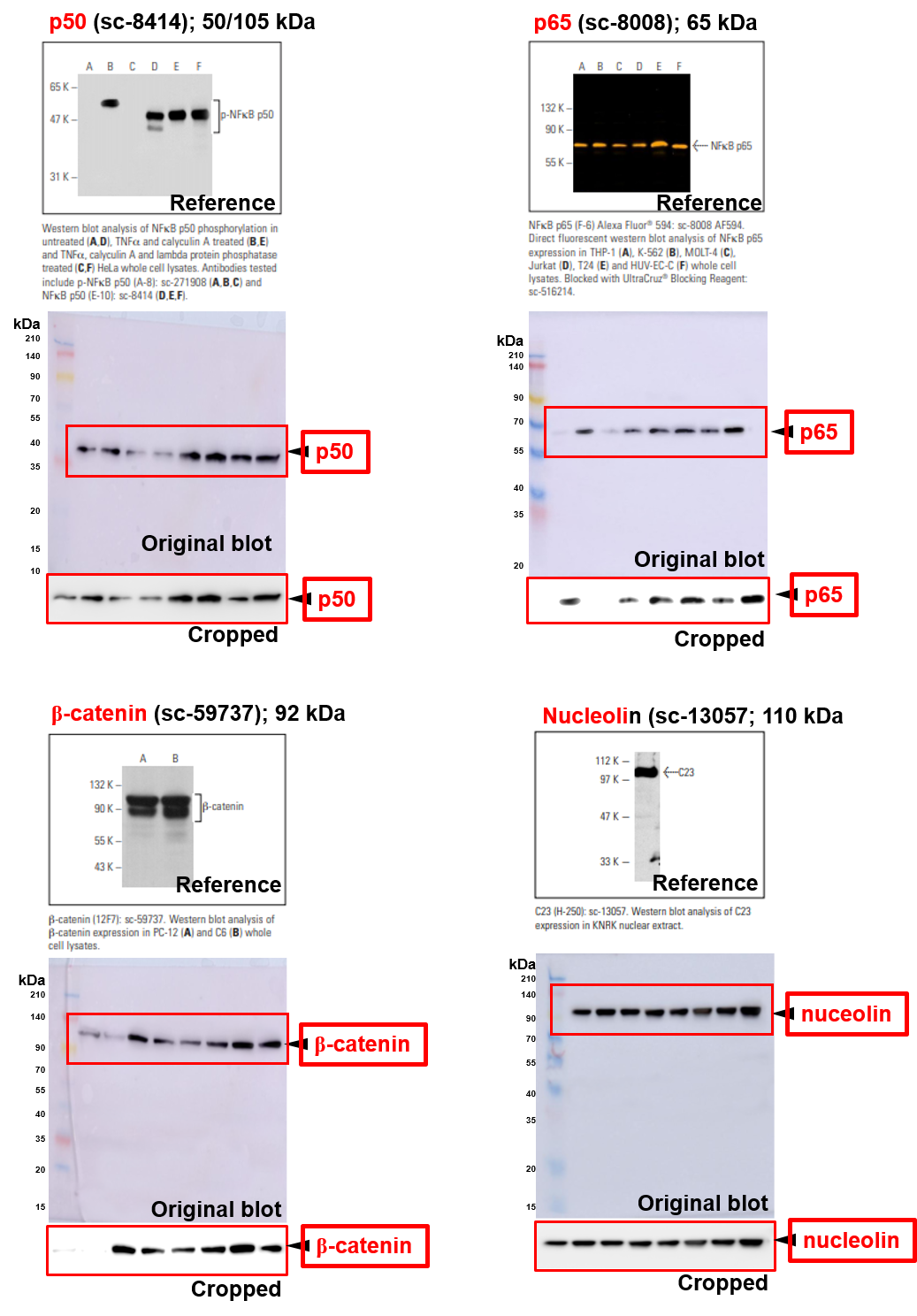


**Figure S8.** Uncropped images of Figure 7A.


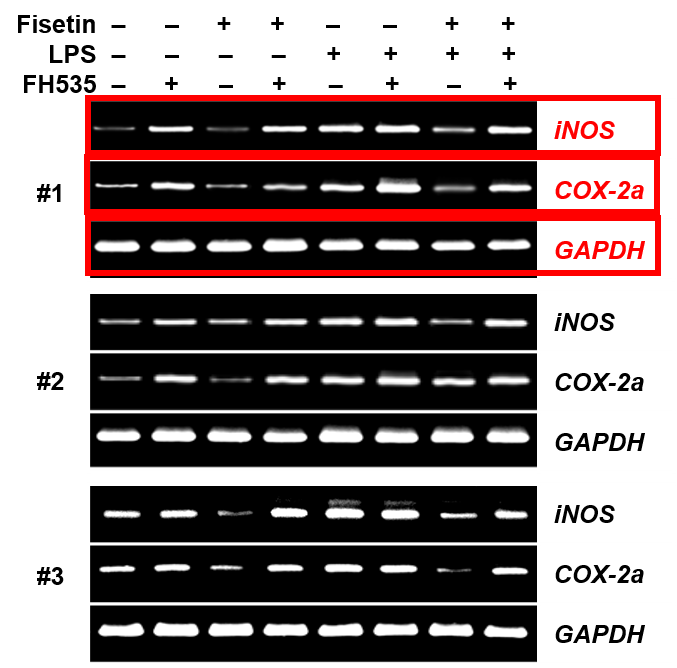


**Figure S9.** Triplicated images of Figure 8C.
